# A partially penalty immersed Crouzeix-Raviart finite element method for interface problems

**DOI:** 10.1186/s13660-017-1461-5

**Published:** 2017-08-14

**Authors:** Na An, Xijun Yu, Huanzhen Chen, Chaobao Huang, Zhongyan Liu

**Affiliations:** 1 School of Mathematics and Statistics, Shandong Normal University, Jinan, 250014 China; 2 Laboratory of Computational Physics, Institute of Applied Physics and Computational Mathematics, Beijing, 100088 China; 3 Applied and Computational Mathematics Division, Beijing Computational Science Research Center, Beijing, 100193 China

**Keywords:** elliptic interface problems, discontinuous coefficients, partially penalty, immersed finite element method, Crouzeix-Raviart element, optimal-order error estimates

## Abstract

The elliptic equations with discontinuous coefficients are often used to describe the problems of the multiple materials or fluids with different densities or conductivities or diffusivities. In this paper we develop a partially penalty immersed finite element (PIFE) method on triangular grids for anisotropic flow models, in which the diffusion coefficient is a piecewise definite-positive matrix. The standard linear Crouzeix-Raviart type finite element space is used on non-interface elements and the piecewise linear Crouzeix-Raviart type immersed finite element (IFE) space is constructed on interface elements. The piecewise linear functions satisfying the interface jump conditions are uniquely determined by the integral averages on the edges as degrees of freedom. The PIFE scheme is given based on the symmetric, nonsymmetric or incomplete interior penalty discontinuous Galerkin formulation. The solvability of the method is proved and the optimal error estimates in the energy norm are obtained. Numerical experiments are presented to confirm our theoretical analysis and show that the newly developed PIFE method has optimal-order convergence in the $L^{2}$ norm as well. In addition, numerical examples also indicate that this method is valid for both the isotropic and the anisotropic elliptic interface problems.

## Introduction

The elliptic equations with discontinuous coefficients are often used to describe phenomena appearing in material sciences and fluid dynamics when there are two or more distinct materials or fluids with different densities or conductivities or diffusivities. Since the solutions of these interface problems are required to satisfy interface jump conditions from conservation laws, it is difficult to find the exact solutions and construct high accuracy numerical methods. In addition, if the interface is smooth enough, then the solution of the interface problem is also smooth in individual regions where the coefficient is smooth. But due to the jump of the coefficient along the interface, the global regularity of the solution is usually low and the solution belongs to $H^{1+\alpha}(\Omega)$, $0 \leq\alpha< 1$. Therefore, it is difficult to achieve high accuracy by using standard finite element methods. Several articles are devoted to developing methods to solve these problems, such as fitted finite element methods [1–3], proposed by Chen and Zou in [4], discontinuous Galerkin methods [5, 6], weak Galerkin finite element methods [7, 8], proposed by Wang and Ye in [9–11], immersed boundary methods [12, 13], the extended/generalized finite element methods [14], immersed interface methods, which are a kind of finite difference methods, proposed by LeVeque and Li in [15], and immersed finite volume methods [16, 17].

In recent years, the immersed finite element (IFE) methods have been studied and found to be very effective for solving elliptic interface problems. The method was proposed on the uniform Cartesian triangular grids in [18, 19], according to the above mentioned immersed interface methods. Approximation capabilities of the nonconforming IFE spaces were studied in [20] and the convergence analysis of the IFE solutions was developed in [21, 22]. But we realize that it is very difficult to achieve an optimal-order $H^{1}$-norm error estimate due to the strong nonconformity. So, for anisotropic elliptic interface problems, we presented the partially penalty IFE method in [23] by adding two penalty terms on the common edges of the adjacent interface elements to restrict the function jumps, and then deriving the optimal-order error estimates. The numerical experiments verify our theoretical results. Moreover, this idea of partially penalization can also be seen in [24], which is on rectangular grids for solving isotropic elliptic interface problems. And there are other developed forms [25–27]. In addition, the penalty idea exists in other unfitted methods, too. In [28], a ghost penalty is added in unfitted finite element methods to recover the condition number of the stiffness matrix. And an unfitted method based on the symmetric interior penalty discontinuous Galerkin method was proposed to discretize elliptic interface problems in [29]. Moreover, there are other forms of IFE methods, such as the symmetric and consistent IFE method [30] and the augmented IFE method [31, 32].

Recently, Kwak *et al.* [33] proposed an immersed finite element method based on piecewise linear Crouzeix-Raviart type polynomials on a uniform triangular grid. In this reference, the authors use the integral averages on the edges as degrees of freedom to weaken their nonconformity on the common edge of two adjacent interface elements. Zhang, in his thesis [34], proposed the nonconforming rotated $Q_{1}$ IFE method based on rectangular grids by using both integral averages and midpoint values on the edges as degrees of freedom.

However, the optimal convergence is still not very easily obtained. And inspired by the work of Ji *et al.* [28], a penalty on interface edges is added to ensure that the condition number of the resulting linear system is independent of how the interface cuts through the mesh. In this paper, we develop the partially penalty immersed finite element (PIFE) method with the Crouzeix-Raviart type polynomial spaces to solve the anisotropic flow models in which the diffusion coefficient is a piecewise definite-positive matrix, although we consider isotropic elliptic problems with piecewise scalar coefficients in major length for simplicity. For the anisotropic elliptic interface problems, the construction of the Crouzeix-Raviart type IFE spaces is given in our paper [35]. We prove that the piecewise Crouzeix-Raviart type polynomials satisfying the jump conditions on two types of interface elements can be uniquely determined by integral averages on the edges as degrees of freedom. Then we construct the Crouzeix-Raviart type IFE spaces on interface elements and give the partially penalty immersed Crouzeix-Raviart finite element schemes. We prove the solvability of the method and obtain its optimal convergence analysis. Numerical experiments show that our method is valid not only for isotropic elliptic interface problems but also for anisotropic elliptic interface problems.

The rest of the paper is organized as follows. In the next section, some preliminaries and notations are introduced. In Section 3, we construct IFE spaces based on the Crouzeix-Raviart elements on triangular grids and investigate properties of these nonconforming IFE spaces. In Section 4, we are devoted to defining the PIFE method based on the Crouzeix-Raviart type IFE spaces and prove the solvability of the method. Then we carry out the optimal convergence analysis. In Section 6, some numerical experiments are performed to indicate the optimal-order convergence of our PIFE method for both isotropic and anisotropic elliptic interface problems. Finally, we conclude in the last section.

## Preliminaries and notations

**Figure 1:**
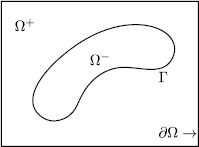
**The geometry of the computational domain** **Ω.**

Consider the second order elliptic interface problem:

2.1 $$ \textstyle\begin{cases} (\mathrm{a})\quad {-}\nabla\cdot(\beta(\mathbf {x})\nabla u)=f,\quad \mathbf {x}\in\Omega, \\ (\mathrm{b})\quad u=0,\quad \mathbf {x}\in\partial\Omega, \end{cases} $$

where $\beta(\mathbf {x})$ is a discontinuous coefficient, $f\in L^{2}(\Omega )$, $\mathbf {x}=(x,y)$. The convex polygonal domain $\Omega\subset\mathbf {R}^{2}$ consists of $\Omega^{+}$ and $\Omega^{-}$, $\Omega^{+}\cap\Omega^{-}=\emptyset$; see Figure 1 (cited from [23]) for illustration. Assume that the interface $\Gamma=\partial\Omega^{-}\subset\Omega$ is smooth enough ($C^{2}$) and the solution *u* satisfies the following jump conditions across the interface Γ:

2.2 $$ [u]=0 \quad \text{and} \quad \bigl[\beta(\mathbf {x})\nabla u \cdot \mathbf {n}_{\Gamma} \bigr]=0, $$

where the jump value $[u]=u|_{\Omega^{-}}-u|_{\Omega^{+}}$ and $\mathbf {n}_{\Gamma}$ denotes the unit normal vector of Γ pointing from $\Omega^{-}$ to $\Omega^{+}$.

For the sake of simplicity and convenience, in the following analysis, we assume $\beta(\mathbf {x})$ is a piecewise constant function defined by

$$ \beta(\mathbf {x})=\beta= \textstyle\begin{cases} \beta^{-},& \mathbf {x}\in\Omega^{-}, \\ \beta^{+},& \mathbf {x}\in\Omega^{+}, \end{cases} $$

where $\beta^{-} >0$, $\beta^{+}>0$.

Of course, the coefficient $\beta(\mathbf {x})$ can also be a symmetric definite-positive matrix as

2.3 $$ \beta(\mathbf {x})=\mathbb{B}^{l}=\left ( \textstyle\begin{array}{@{}c@{\quad}c@{}} m^{l}& s^{l}\\ s^{l}&n^{l} \end{array}\displaystyle \right ),\quad \mathbf {x}\in\Omega^{l}, l=\pm, $$

and the corresponding analysis can be performed similarly. In the last section, we also conduct numerical examples for this case.

Let $\mathcal{T}_{h}=\{T\}$ be the usual regular triangulation of the domain Ω. $\mathcal{T}_{h}^{i}$ and $\mathcal{T}_{h}^{n}$ denote the collection of interface elements and the collection of the non-interface elements, respectively. It is also called a non-interface element if Γ intersects with this triangle but does not separate its interior into two nontrivial subsets. Assume that the interface meets the edges of an interface element at no more than two intersections. Such an assumption is reasonable if the step size is sufficiently small.

Let $\mathcal{E}_{h}$ be the collection of all edges in the triangulation $\mathcal{T}_{h}=\mathcal{T}_{h}^{i} \cup\mathcal{T}_{h}^{n}$. $\mathcal{E}^{\circ}_{h}$ and $\mathcal{E}^{b}_{h}$ denote the interior edges and boundary edges, respectively. Moreover, the sets of the interface edges and non-interface edges are denoted by $\mathcal {E}^{i}_{h}$ and $\mathcal{E}^{n}_{h}$. Obviously, here we have $\mathcal {E}_{h}=\mathcal{E}^{\circ}_{h}\cup\mathcal{E}^{b}_{h}$ and also $\mathcal {E}_{h}=\mathcal{E}^{i}_{h}\cup\mathcal{E}^{n}_{h}$.

In the following, we define the jump and average values of a function *u* on the edges. For every interior edge $e\in\mathcal{E}^{\circ}_{h}$,

$$[u]_{e}=u|_{T_{e,1}}-u|_{T_{e,2}},\qquad \{u \}_{e}=\frac {1}{2}(u|_{T_{e,1}}+u|_{T_{e,2}}), $$

where $T_{e,1}$ and $T_{e,2}$ are the two elements sharing the common edge *e* and the unit normal vector of *e* is assumed to point from $T_{e,1}$ to $T_{e,2}$. For every boundary edge $e\in\mathcal{E}^{b}_{h}$,

$$[u]_{e}=\{u\}_{e}=u|_{T_{e}}, $$

where $T_{e}$ is the element such that $e\in\mathcal{E}^{b}_{h}$ is one of its edges. Usually we omit the subscript in $[\cdot]$ and $\{\cdot\}$ if there is no confusion.

For the analysis, we introduce the following spaces on the whole domain Ω:

$$\begin{aligned}& \widetilde{H}^{2}(\Omega)=\bigl\{ u\in H^{1}(\Omega):u\in H^{2}\bigl(\Omega ^{l}\bigr), l=\pm\bigr\} , \\& \widetilde{H}^{2}_{\mathrm{int}}(\Omega)=\bigl\{ u\in H^{1}( \Omega): u|_{\Omega ^{l}}\in H^{2}\bigl(\Omega^{l}\bigr), l= \pm, [\beta\nabla u\cdot \mathbf {n}]=0 \text{ across } \Gamma\bigr\} , \end{aligned}$$

and for any $u\in\widetilde{H}^{2}(\Omega)$,

2.4 $$ \|u\|_{\widetilde{H}^{2}(\Omega)}^{2}=\|u\|_{H^{2}(\Omega^{+})}^{2}+ \|u\| _{H^{2}(\Omega^{-})}^{2}, $$

where $H^{1}(\Omega)=W^{1,2}(\Omega)$ and $H^{2}(\Omega ^{l})=W^{2,2}(\Omega^{l})$ are the usual Sobolev spaces.

For every interface element $T\in\mathcal{T}_{h}^{i}$, we also introduce the following spaces:

$$\begin{aligned}& \widetilde{H}^{2}(T)=\bigl\{ u: u|_{T \cap\Omega^{l}}\in H^{2} \bigl(T \cap\Omega ^{l}\bigr), l=\pm\bigr\} , \\& \widetilde{H}^{2}_{\mathrm{int}}(T)=\bigl\{ u\in H^{1}(T): u|_{T \cap\Omega^{l}}\in H^{2}\bigl(T \cap\Omega^{l}\bigr), l= \pm, [\beta\nabla u\cdot \mathbf {n}]=0 \text{ across } \Gamma\cap T\bigr\} , \end{aligned}$$

and for any $u\in\widetilde{H}^{2}(T)$,

2.5 $$ \|u\|^{2}_{2,T}=\|u\|^{2}_{2,T \cap\Omega^{+}}+ \|u\|^{2}_{2,T \cap\Omega ^{-}},\qquad |u|^{2}_{2,T}=|u|^{2}_{2,T \cap\Omega^{+}}+|u|^{2}_{2,T \cap\Omega^{-}}, $$

where $\|\cdot\|_{2,T\cap\Omega^{l}}$ is the norm of $H^{2}(T\cap\Omega ^{l})$, $l=\pm$.

## The Crouzeix-Raviart type IFE space and its properties

In this section, we introduce the local Crouzeix-Raviart type basis functions for both the non-interface elements and the interface elements, define the IFE spaces over the whole domain, and then investigate properties of the nonconforming Crouzeix-Raviart type IFE spaces.

### Construction of immersed Crouzeix-Raviart finite element spaces

In this subsection, we develop the nonconforming IFE spaces with integral averages on the edges as degrees of freedom. To make sure that the flux jump conditions can be weakly enforced on the smooth interface, we derive the IFE functions on usual elements instead of the reference elements used in [33]. The standard nonconforming linear Crouzeix-Raviart functions are used on non-interface elements. On interface elements, these functions are locally modified to satisfy the jump conditions.

For a non-interface element $T\in\mathcal{T}_{h}^{n}$, we simply use the standard linear Crouzeix-Raviart type polynomials as local basis functions, and use $\overline{S}_{h}(T)$ to denote spaces spanned by the three basis functions on *T*,

$$ \overline{S}_{h}(T)=\operatorname{span}\biggl\{ \phi_{i}\in P_{1}(T); \frac {1}{|e_{j}|} \int_{e_{j}}\phi_{i} \,ds=\delta_{ij}, i,j=1,2,3\biggr\} , $$

where $e_{j}$, $j=1, 2, 3$ are three edges of *T* and *δ* is the Kronecker function.

For the space $\overline{S}_{h}(T)$ on non-interface element $T\in \mathcal{T}_{h}^{n}$, we have the well-known approximation property as follows [36, 37]:

3.1 $$ \|u-I_{T}u\|_{L^{2}(T)}+h\|u-I_{T}u \|_{H^{1}(T)}\leq Ch^{2} \|u\|_{H^{2}(T)},\quad \forall u \in{H}^{2}(T), $$

where $I_{T}: H^{2}(T)\rightarrow\overline{S}_{h}(T)$ is the interpolation operator defined by

3.2 $$ \int_{e_{j}}I_{T}u \,ds= \int_{e_{j}}u\,ds,\quad j=1,2,3. $$

To construct the local immersed Crouzeix-Raviart finite element spaces on interface elements, we consider a typical triangle $T\in\mathcal {T}_{h}^{i}$, in which the three vertices are $A_{1}(0,0)$, $A_{2}(h,0)$ and $A_{3}(0,h)$. We assume that the interface curve Γ intersects *T* at two different points *D* and *E*. And the segment $\overline{DE}$ separates *T* into two subsets $T^{+}$ and $T^{-}$ with $T=T^{+}\cup T^{-}\cup \overline{DE}$.

**Figure 2:**
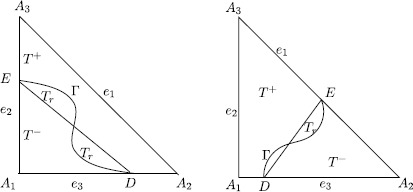
**Type-1** **(left) and Type-2** **(right) interface triangular elements.**

There are two types of interface elements depending on the location of the intersection points *D* and *E*. We call an element *T* a Type-1 interface element if Γ intersects with *T* at two square edges, or a Type-2 interface element if Γ intersects with *T* at a square edge and a bevel edge. See Figure 2 for an illustration of the two different types of interface elements, where, without loss of generality, we assume that intersection points *D*, *E* satisfy

$$D=(ah,0)\in\overline{A_{1}A_{2}}, \qquad E=(0,bh)\in \overline{A_{1}A_{3}}, $$

for a Type-1 interface element with $0< a, b\leq1$, and

$$D=(ah,0)\in\overline{A_{1}A_{2}}, \qquad E= \bigl((1-b)h,bh \bigr)\in \overline{A_{2}A_{3}}, $$

for a Type-2 interface element with $0\leq a<1$, $0< b<1$.

There is a small region in these two types of interface elements,

$$T_{r}=T\backslash\bigl(\Omega^{+}\cap T^{+}\bigr)\backslash\bigl(\Omega^{-} \cap T^{-}\bigr) $$

whose area is of order $O(h^{3})$ since the interface is a $C^{2}$ curve and the interface $\Gamma\cap T$ is perturbed in a magnitude of $O(h^{2})$ [4]. Therefore, such a perturbation will only affect the solution and the interpolation function to an order of $O(h^{2})$, which will not impact on the convergence accuracy of the method whose approximation spaces are selected as piecewise linear polynomials in this paper.

On each of these interface triangular elements, $T\in\mathcal {T}_{h}^{i}$, for given values $V_{i}$, $i=1, 2, 3$, the piecewise linear function *ϕ* can be defined by

3.3 $$ {\phi}(\mathbf {x})= \textstyle\begin{cases} {{\phi}}^{+}(\mathbf {x})=a_{0}+b_{0}x+c_{0}y, & \mathbf {x}\in{T}^{+},\\ {{\phi}}^{-}(\mathbf {x})=a_{1}+b_{1}x+c_{1}y, & \mathbf {x}\in{T}^{-}, \end{cases} $$

satisfying

3.4 $$ \textstyle\begin{cases} (\mathrm{a})\quad \overline{{\phi}_{e_{i}}}=V_{i}, \quad i=1, 2, 3, \\ (\mathrm{b})\quad {\phi}^{+}(D)={\phi}^{-}(D), \\ (\mathrm{c})\quad {\phi}^{+}(E)={\phi}^{-}(E), \\ (\mathrm{d})\quad (\beta^{+}\nabla{\phi}^{+} -\beta^{-}\nabla{\phi}^{-})\cdot \mathbf {n}_{\overline{DE}}=0, \end{cases} $$

where $\overline{\phi_{e_{i}}}$, $i=1,2,3$ denote integral averages of *ϕ* on the edges $e_{i}$, *i.e.*, $\overline{\phi_{e_{i}}}=\frac{1}{|e_{i}|}\int_{e_{i}}\phi \,ds$, and $\mathbf {n}_{\overline{DE}}$ is a unit normal vector to $\overline{DE}$.

As for the piecewise linear function *ϕ* defined above, we have the following results:

#### Theorem 3.1

*For arbitrary diffusion coefficient* *β* *and interface location*, *the piecewise linear function* ${\phi}(\mathbf {x})$ *on the interface element* $T\in\mathcal{T}_{h}^{i}$ *defined by* (3.3) *and* (3.4) *is uniquely determined by the given three values* $V_{1}$, $V_{2}$, $V_{3}$.

#### Proof

We shall prove the theorem on Type-1 and Type-2 interface elements. In both cases, it is proved by involving $a_{i}$, $b_{i}$, $c_{i}$, $i=0,1$ as unknowns into a linear system and then showing its coefficient matrix is non-singular for arbitrary interface location, reflected by *a* and *b*.

Firstly, we consider the Type-1 interface elements, where $D=(ah,0)$, $E=(0,bh)$ with $0< a, b\leq1$. By the definition of $\overline{{\phi}_{e_{i}}}$ and (3.4a) we obtain, through a simple calculation, the first three equations with respect to unknowns,

3.5 $$ \begin{aligned} &a_{0}+\frac{h}{2}b_{0}+ \frac{h}{2}c_{0}=V_{1}, \\ & \biggl(a_{1}+\frac{bh}{2}c_{1} \biggr)b+ \biggl[a_{0}+\frac{(1+b)h}{2}c_{0} \biggr](1-b) =V_{2}, \\ & \biggl(a_{1}+\frac{ah}{2}b_{1} \biggr)a+ \biggl[a_{0}+\frac{(1+a)h}{2}b_{0} \biggr](1-a)=V_{3}. \end{aligned} $$

The continuity conditions (3.4b) and (3.4c) imply the fourth and fifth equations,

3.6 $$ \begin{aligned} &a_{0}+b_{0}(ah)=a_{1}+b_{1}(ah), \\ &a_{0}+c_{0}(bh)=a_{1}+c_{1}(bh). \end{aligned} $$

The last equation is derived from the flux continuity condition (3.4d) along $\overline{DE}$,

3.7 $$ (b_{0}-\rho b_{1})\cdot(bh)+(c_{0}- \rho c_{1})\cdot(ah)=0, $$

where we used $\mathbf {n}_{\overline{DE}}=(bh,ah)/\sqrt{(bh)^{2}+(ah)^{2}}$ and $\rho=\beta^{-}/\beta^{+}$.

We summarize these six equations and reformulate them into the following matrix form:

3.8 $$ \mathbb{A} \mathbb{Y}=\mathbb{F}, $$

where $\mathbb{Y}=(a_{0},b_{0},c_{0},a_{1},b_{1},c_{1})^{T}$, $\mathbb{F}=(V_{1},V_{2},V_{3},0,0,0)^{T}$ and the coefficient matrix

$$ \mathbb{A}=\left ( \textstyle\begin{array}{@{}c@{\quad}c@{\quad}c@{\quad}c@{\quad}c@{\quad}c@{}} 1 & \frac{1}{2}h & \frac{1}{2}h & 0 & 0 & 0 \\ 1-b & 0 & \frac{1}{2}(1-{b}^{2})h & b & 0 & \frac {1}{2}{b}^{2}h \\ 1-a & \frac{1}{2}(1-{a}^{2})h & 0 & a & \frac{1}{2}{a}^{2}h & 0 \\ 1 & ah & 0 & -1 & -ah & 0 \\ 1 & 0 & bh & -1 & 0 & -bh \\ 0 & bh & ah & 0 & -\rho bh & -\rho ah \end{array}\displaystyle \right ). $$

In the following we will show that the coefficient matrix is non-singular or its determinant is non-zero. Following a tedious calculation, we have

3.9 $$\begin{aligned} \operatorname{det}(\mathbb{A})&=\frac{1}{4}h^{4} \bigl[a{b}^{2}\cdot b+a^{2}b \cdot a+b(1-ab)\cdot\rho b+a(1-ab)\cdot\rho a\bigr] \\ &=\frac{1}{4}h^{4}\bigl(a^{2}+b^{2}\bigr) \cdot\bigl[\rho(1-ab)+ab\bigr]. \end{aligned}$$

By $0< a, b\leq1$, we have $0< ab<1$. Then $\rho>0$ implies that $\operatorname{det}(\mathbb{A})>0$ for this case.

For the Type-2 interface elements, where $D=(ah,0)$, $E= ((1-b)h,bh )$ with $0\leq a<1$, $0< b<1$, by the definition of $\overline{{\phi }_{e_{i}}}$ and (3.4a), we derive the first three equations similarly to the derivation of (3.5),

3.10 $$ \begin{aligned} &(1-b) \biggl[a_{0}+b_{0} \frac{(1-b)h}{2}+c_{0}\frac{(1+b)h}{2} \biggr] +b \biggl[a_{1}+b_{1}\biggl(1-\frac{b}{2} \biggr)h+c_{1}\frac{bh}{2} \biggr]=V_{1}, \\ &a_{0}+c_{0}\frac{h}{2} =V_{2}, \\ &a \biggl(a_{0}+b_{0}\frac{ah}{2} \biggr)+(1-a) \biggl[a_{1}+b_{1}\frac {(1+a)h}{2} \biggr]=V_{3}. \end{aligned} $$

The continuity conditions (3.4b) and (3.4c) imply two equations as below,

3.11 $$ \begin{aligned} &a_{0}+b_{0}(ah)=a_{1}+b_{1}(ah), \\ &a_{0}+b_{0}(1-b)h+c_{0}(bh)=a_{1}+b_{1}(1-b)h+c_{1}(bh). \end{aligned} $$

The last equation is obtained from the flux continuity condition (3.4d),

3.12 $$ (b_{0}-\rho b_{1})\cdot(bh)+(c_{0}- \rho c_{1})\cdot(a+b-1)h=0, $$

where we used $\mathbf {n}_{\overline{DE}}= (bh,(a+b-1)h )/\sqrt {b^{2}h^{2}+(a+b-1)^{2}h^{2}}$ and $\rho=\beta^{-}/\beta^{+}$.

We summarize these six equations and write in the matrix form:

3.13 $$ \mathbb{A} \mathbb{Y}=\mathbb{F}, $$

where $\mathbb{Y}=(a_{0},b_{0},c_{0},a_{1},b_{1},c_{1})^{T}$, $\mathbb{F}=(V_{1},V_{2},V_{3},0,0,0)^{T}$ and the coefficient matrix

$$ \mathbb{A}=\left ( \textstyle\begin{array}{@{}c@{\quad}c@{\quad}c@{\quad}c@{\quad}c@{\quad}c@{}} 1-b & \frac{(1-b)^{2}}{2}h & \frac{1-b^{2}}{2}h & b & (1-\frac {b}{2})bh & \frac{b^{2}}{2}h \\ 1 & 0 & \frac{h}{2} & 0 & 0 & 0 \\ a & \frac{a^{2}}{2}h & 0 & 1-a & \frac{1-a^{2}}{2}h & 0 \\ 1 & ah & 0 & -1 & -ah & 0 \\ 1 & (1-b)h & bh & -1 & -(1-b)h & -bh \\ 0 & bh & (a+b-1)h & 0 & -\rho bh & -\rho(a+b-1)h \end{array}\displaystyle \right ). $$

In the following we will prove that the coefficient matrix is non-singular. Following a tedious calculation, we have

3.14 $$\begin{aligned} \operatorname{det}(\mathbb{A}) =&\frac{1}{4}h^{4} \bigl\{ b^{2}(1-a)\cdot b+b(1-a) (a+b-1)\cdot(a+b-1) \\ &{} +b \bigl[1-b(1-a) \bigr] \cdot\rho b+ \bigl[b(1-a)^{2}+b-(1-a) \bigl(1+b^{2}\bigr) \bigr]\cdot\rho(a+b-1) \bigr\} \\ =&\frac{1}{4}h^{4} \bigl\{ b^{3}(1-a)+b(1-a) (1-a-b)^{2}+\rho b^{2} \bigl[1-b(1-a) \bigr] \\ &{} +\rho(1-a-b) (1-a) \bigl(1+b^{2}\bigr)-\rho b(1-a-b) \bigl[(1-a)^{2}+1 \bigr] \bigr\} . \end{aligned}$$

To simplify the proof, we let $1-a=tb$ with $t>0$, then $\operatorname{det}(\mathbb{A})$ is rewritten as

$$\begin{aligned} \operatorname{det}(\mathbb{A}) =& \frac{1}{4}h^{4} \bigl[tb^{4}+t(t-1)^{2}b^{4}+\rho b^{2} \bigl(1-tb^{2}\bigr) \\ &{} +\rho t(t-1)b^{2}\bigl(1+b^{2}\bigr)- \rho(t-1)b^{2}\bigl(t^{2}b^{2}+1\bigr) \bigr] \\ =& \frac{1}{4}h^{4}b^{2} \bigl[tb^{2}+t(t-1)^{2}b^{2}- \rho tb^{2}+\rho t(t-1)b^{2}-\rho t^{2}(t-1)b^{2} \\ &{} +\rho+\rho t(t-1)-\rho(t-1) \bigr] \\ =&\frac{1}{4}h^{4}b^{2} \bigl[t(1-\rho) \bigl(t^{2}-2t+2\bigr)b^{2}+\rho\bigl(t^{2}-2t+2 \bigr) \bigr] \\ =&\frac{1}{4}h^{4}b^{2} \bigl\{ \bigl(t^{2}-2t+2 \bigr)\cdot \bigl[t(1-\rho)b^{2}+\rho \bigr] \bigr\} \\ =&\frac{1}{4}h^{4}b^{2} \bigl\{ \bigl[(t-1)^{2}+1 \bigr]\cdot \bigl[tb^{2}+ \bigl(1-tb^{2}\bigr)\rho \bigr] \bigr\} . \end{aligned}$$

By $0\leq a<1$, we have $0< tb=1-a\leq1$. Also by $0< b<1$, we derive $0< tb^{2}<1$ and $1-tb^{2}>0$. Therefore $\rho>0$ implies $\operatorname{det}(\mathbb{A})>0$ in this case, which completes the proof. □

#### Remark 3.2

Theorem 3.1 tells us that on each interface element, the piecewise linear function defined by (3.3) and (3.4) is continuous across $\overline{DE}$ and uniquely determined by its integral averages on the edges as degrees of freedom.

Then, for an interface element $T\in\mathcal{T}_{h}^{i}$, we can define the local Crouzeix-Raviart type IFE space $\widehat{S}_{h}( T)$ by

3.15 $$\begin{aligned}& \widehat{S}_{h}( T)= \operatorname{span} \bigl\{ \hat{ \phi_{i}}(\mathbf {x}); \hat{\phi_{i}}(\mathbf {x}), i=1,2,3 \text{ are uniquely determined by } (3.3) \text{ and } (3.4)\bigr\} , \\& \hat{\phi_{1}}(\mathbf {x})= \textstyle\begin{cases} {\hat{\phi}_{1}}^{+}(\mathbf {x})=\mathbb{A}^{-1}(1,1)+\mathbb {A}^{-1}(2,1)x+\mathbb{A}^{-1}(3,1)y, & \mathbf {x}\in{T}^{+}, \\ {\hat{\phi}_{1}}^{-}(\mathbf {x})=\mathbb{A}^{-1}(4,1)+\mathbb {A}^{-1}(5,1)x+\mathbb{A}^{-1}(6,1)y, & \mathbf {x}\in{T}^{-}, \end{cases}\displaystyle \end{aligned}$$

3.16 $$\begin{aligned}& \hat{\phi_{2}}(\mathbf {x})= \textstyle\begin{cases} {\hat{\phi}_{2}}^{+}(\mathbf {x})=\mathbb{A}^{-1}(1,2)+\mathbb {A}^{-1}(2,2)x+\mathbb{A}^{-1}(3,2)y, & \mathbf {x}\in{T}^{+}, \\ {\hat{\phi}_{2}}^{-}(\mathbf {x})=\mathbb{A}^{-1}(4,2)+\mathbb {A}^{-1}(5,2)x+\mathbb{A}^{-1}(6,2)y, & \mathbf {x}\in{T}^{-}, \end{cases}\displaystyle \end{aligned}$$

3.17 $$\begin{aligned}& \hat{\phi_{3}}(\mathbf {x})= \textstyle\begin{cases} {\hat{\phi}_{3}}^{+}(\mathbf {x})=\mathbb{A}^{-1}(1,3)+\mathbb {A}^{-1}(2,3)x+\mathbb{A}^{-1}(3,3)y, & \mathbf {x}\in{T}^{+}, \\ {\hat{\phi}_{3}}^{-}(\mathbf {x})=\mathbb{A}^{-1}(4,3)+\mathbb {A}^{-1}(5,3)x+\mathbb{A}^{-1}(6,3)y, & \mathbf {x}\in{T}^{-}, \end{cases}\displaystyle \end{aligned}$$

where ${\mathbb{A}}^{-1}$ denotes the inverse matrix of $\mathbb{A}$ that is derived in the proof of Theorem 3.1.

#### Remark 3.3

Theorem 3.1 also holds if the coefficient $\beta(\mathbf {x})$ is a symmetric definite-positive matrix defined by (2.3) and its proof is provided in another paper [35].

Finally, we conclude this subsection by defining the global immersed Crouzeix-Raviart type finite element spaces ${S}_{h}(\Omega)$ and ${S}_{0h}(\Omega)$ on the whole domain Ω as

3.18 $$ \begin{aligned} &{S}_{h}(\Omega)= \biggl\{ \phi_{h}\in L^{2}(\Omega): \phi_{h}|_{T\in\mathcal{T}_{h}^{n}} \in \overline{S}_{h}(T) \mbox{ and } \phi_{h}|_{T\in\mathcal{T}_{h}^{i}} \in\widehat{S}_{h}(T); \\ &\hphantom{{S}_{h}(\Omega)={}}\text{if } T_{1}\cap T_{2}=e, \text{then } \int_{e} \phi_{h}|_{T_{1}} \,ds= \int_{e} \phi_{h}|_{T_{2}} \,ds\biggr\} , \\ &{S}_{0h}(\Omega)=\biggl\{ \phi_{h}\in{S}_{h}( \Omega): \int_{e}\phi_{h} \,ds=0, \forall e \in \mathcal{E}^{b}_{h}\biggr\} . \end{aligned} $$

### Properties of the Crouzeix-Raviart type IFE spaces

In this subsection, we present several properties for the local IFE space $\widehat{S}_{h}( T)$ on the interface element $T\in\mathcal {T}_{h}^{i}$ and the global IFE space ${S}_{0h}(\Omega)$ on the domain Ω.

Although for Crouzeix-Raviart type IFE functions in $\widehat{S}_{h}( T)$ the flux jump condition is enforced on the line segment $\overline {DE}$, they actually satisfy a weak flux jump condition along the actual interface curve $\Gamma\cap T$, which is stated in the following lemma.

#### Lemma 3.4

*For an interface triangle* $T\in\mathcal{T}_{h}^{i}$, *each linear function* $\phi\in \widehat{S}_{h}(T)$ *satisfies the flux jump condition on* $\Gamma\cap T$ *in the following weak sense*:

3.19 $$ \int_{\Gamma\cap T}\bigl(\beta^{-} \nabla\phi^{-}-\beta^{+}\nabla\phi ^{+}\bigr) \cdot \mathbf {n}_{\Gamma}\,ds=0. $$

#### Proof

Let *ϕ* be a function in $\widehat{S}_{h}(T)$. By Green’s formula we have

$$\begin{aligned}& \int_{\Gamma\cap T}\bigl(\beta^{-}\nabla{\phi}^{-} -\beta^{+}\nabla{\phi}^{+}\bigr)\cdot \mathbf {n}_{\Gamma} \,ds+ \int_{{\overline{DE}}}\bigl(\beta^{-}\nabla{\phi}^{-} -\beta^{+}\nabla{\phi}^{+}\bigr)\cdot \mathbf {n}_{\overline{DE}}\,ds \\& \quad = \int_{T_{r}}\operatorname{div}\bigl(\beta^{-}\nabla{ \phi}^{-} -\beta^{+}\nabla{\phi}^{+}\bigr) \,d\mathbf {x}\\& \quad =0. \end{aligned}$$

Combining the equality above with the flux continuity of *ϕ* along $\overline{DE}$

$$\int_{{\overline{DE}}}\bigl(\beta^{-}\nabla{\phi}^{-} -\beta^{+}\nabla{\phi}^{+}\bigr)\cdot \mathbf {n}_{\overline{DE}} \,ds=0, $$

we reach the conclusion. □

It is well known that the trace inequalities are important for the finite element analysis. So here we present the trace inequalities stated on p.23 of [38],

3.20 $$\begin{aligned}& \Vert v \Vert _{0,e}\leq Ch^{-1/2}\bigl( \Vert v \Vert _{0,T}+h \Vert \nabla v \Vert _{0,T} \bigr), \quad \forall v\in H^{1}(T), \end{aligned}$$

3.21 $$\begin{aligned}& \Vert \nabla v\cdot \mathbf {n}\Vert _{0,e}\leq Ch^{-1/2}\bigl( \Vert \nabla v \Vert _{0,T}+h \bigl\Vert \nabla ^{2} v \bigr\Vert _{0,T}\bigr), \quad \forall v\in H^{2}(T). \end{aligned}$$

Noting that $\overline{S}_{h}(T)\subset H^{2}(T)$, the above two inequalities both hold on the non-interface element $T\in\mathcal {T}_{h}^{n}$. However, $\widehat{S}_{h}(T)$ is a subspace of $H^{1}(T)$ only, not a subspace of $H^{2}(T)$. Therefore, we need to establish a trace inequality similar to (3.21) for the Crouzeix-Raviart type IFE functions on interface elements.

#### Theorem 3.5

*There exists a constant* *C* *depending on the diffusion coefficient* *β* *only*, *such that on any* $T\in\mathcal{T}_{h}^{i}$,

3.22 $$ \|\beta\nabla\phi\cdot \mathbf {n}\|_{0,e}\leq Ch^{-1/2}\|\nabla\phi\| _{0,T}, \quad \forall \phi\in\widehat{S}_{h}(T), $$

*where* *e* *is an interface edge of* *T*, *and* **n** *is the unit outer normal vector of* *T*.

To prove Theorem 3.5, which follows the idea of proof for Theorem 3.3 in [34], we need the lemma below.

#### Lemma 3.6

*There exist two constants* $C_{1}\geq2$, $C_{2}\geq2$, *which are both dependent on the discontinuous coefficient* *β* *but independent of the mesh size* *h* *and the interface location* (*reflected by* *a*, *b*), *such that*, *for every function* *ϕ* *defined by* (3.3), *we have*

3.23 $$ \frac{1}{C_{2}}\bigl( \vert b_{1} \vert + \vert c_{1} \vert \bigr)\leq \vert b_{0} \vert + \vert c_{0} \vert \leq C_{1}\bigl( \vert b_{1} \vert + \vert c_{1} \vert \bigr). $$

#### Proof

We shall prove the lemma on Type-1 and Type-2 interface elements, respectively.

Firstly, we consider the Type-1 interface elements, where $D=(ah,0)$, $E=(0,bh)$ with $0< a, b\leq1$. According to equations (3.6) and (3.7), we have

$$ \left ( \textstyle\begin{array}{@{}c@{}} a_{0}\\ b_{0}\\ c_{0} \end{array}\displaystyle \right )=\left ( \textstyle\begin{array}{@{}c@{\quad}c@{\quad}c@{}} 1&ah&0\\ 1&0&bh\\ 0&bh&ah \end{array}\displaystyle \right )^{-1} \left ( \textstyle\begin{array}{@{}c@{\quad}c@{\quad}c@{}} 1&ah&0\\ 1&0&bh\\ 0&\rho bh& \rho ah \end{array}\displaystyle \right ) \left ( \textstyle\begin{array}{@{}c@{}} a_{1}\\ b_{1}\\ c_{1} \end{array}\displaystyle \right ). $$

By a tedious calculation, we derive

$$ \left ( \textstyle\begin{array}{@{}c@{}} a_{0}\\ b_{0}\\ c_{0} \end{array}\displaystyle \right )=\frac{1}{a^{2}+b^{2}} \left ( \textstyle\begin{array}{@{}c@{\quad}c@{\quad}c@{}} a^{2}+b^{2} &(1-\rho)ab^{2}h &(1-\rho)a^{2}bh\\ 0 &a^{2}+\rho b^{2} &-(1-\rho)ab\\ 0 &-(1-\rho)ab & b^{2}+\rho a^{2} \end{array}\displaystyle \right ) \left ( \textstyle\begin{array}{@{}c@{}} a_{1}\\ b_{1}\\ c_{1} \end{array}\displaystyle \right ). $$

Then, by using $\rho>0$ and $2ab\leq a^{2}+b^{2}$, we get the inequality for $|b_{0}|$,

$$\begin{aligned} \vert b_{0} \vert &= \biggl\vert \frac{a^{2}+\rho b^{2}}{a^{2}+b^{2}}b_{1} + \frac{-(1-\rho )ab}{a^{2}+b^{2}}c_{1} \biggr\vert \\ &\leq \biggl\vert \frac{a^{2}+\rho b^{2}}{a^{2}+b^{2}} \biggr\vert \cdot \vert b_{1} \vert + \vert 1-\rho \vert \cdot \biggl\vert \frac{ab}{a^{2}+b^{2}} \biggr\vert \cdot \vert c_{1} \vert \\ &\leq\max\{\rho, 1\}\cdot \vert b_{1} \vert + \frac{1}{2} \vert 1-\rho \vert \cdot \vert c_{1} \vert \\ &\leq\max\biggl\{ \rho, 1, \frac{1}{2} \vert 1-\rho \vert \biggr\} \cdot \bigl( \vert b_{1} \vert + \vert c_{1} \vert \bigr). \end{aligned}$$

And in the same way, the inequality for $|c_{0}|$ is derived:

$$ \vert c_{0} \vert \leq\max \biggl\{ \rho, 1, \frac{1}{2} \vert 1-\rho \vert \biggr\} \cdot \bigl( \vert b_{1} \vert + \vert c_{1} \vert \bigr). $$

That is to say, we have

$$ \vert b_{0} \vert + \vert c_{0} \vert \leq2\max \biggl\{ 1, \frac{\beta^{-}}{\beta^{+}}, \frac{1}{2} \biggl\vert 1- \frac{\beta^{-}}{\beta^{+}} \biggr\vert \biggr\} \cdot \bigl( \vert b_{1} \vert + \vert c_{1} \vert \bigr) \triangleq C_{1} \bigl( \vert b_{1} \vert + \vert c_{1} \vert \bigr). $$

If we express $a_{1}$, $b_{1}$, $c_{1}$ by $a_{0}$, $b_{0}$, $c_{0}$ by use of (3.6) and (3.7), then we get the inequalities for $|b_{1}|+|c_{1}|$ similarly:

$$ \vert b_{1} \vert + \vert c_{1} \vert \leq2\max \biggl\{ 1, \frac{\beta^{+}}{\beta^{-}}, \frac{1}{2} \biggl\vert 1- \frac{\beta^{+}}{\beta^{-}} \biggr\vert \biggr\} \cdot \bigl( \vert b_{0} \vert + \vert c_{0} \vert \bigr) \triangleq C_{2} \bigl( \vert b_{0} \vert + \vert c_{0} \vert \bigr). $$

Therefore, we derive the conclusion (3.23) with

$$C_{1}=2\max \biggl\{ 1, \frac{\beta^{-}}{\beta^{+}}, \frac {1}{2} \biggl\vert 1-\frac{\beta^{-}}{\beta^{+}} \biggr\vert \biggr\} , \qquad C_{2}=2\max \biggl\{ 1, \frac{\beta^{+}}{\beta^{-}}, \frac{1}{2} \biggl\vert 1- \frac {\beta^{+}}{\beta^{-}} \biggr\vert \biggr\} . $$

Next we consider the Type-2 interface elements, where $D=(ah,0)$, $E= ((1-b)h,bh )$ with $0\leq a<1$, $0< b<1$.

According to equations (3.11) and (3.12), we have

$$ \left ( \textstyle\begin{array}{@{}c@{}} a_{0}\\ b_{0}\\ c_{0} \end{array}\displaystyle \right )=\left ( \textstyle\begin{array}{@{}c@{\quad}c@{\quad}c@{}} 1&ah&0\\ 1&(1-b)h&bh\\ 0&bh&(a+b-1)h \end{array}\displaystyle \right )^{-1} \left ( \textstyle\begin{array}{@{}c@{\quad}c@{\quad}c@{}} 1&ah&0\\ 1&(1-b)h&bh\\ 0&\rho bh&\rho(a+b-1)h \end{array}\displaystyle \right ) \left ( \textstyle\begin{array}{@{}c@{}} a_{1}\\ b_{1}\\ c_{1} \end{array}\displaystyle \right ). $$

By a tedious calculation, we obtain

$$ \left ( \textstyle\begin{array}{@{}c@{}} a_{0}\\ b_{0}\\ c_{0} \end{array}\displaystyle \right )=\frac{1}{M} \left ( \textstyle\begin{array}{@{}c@{\quad}c@{\quad}c@{}} M &(1-\rho)ab^{2}h &(1-\rho)ab(a+b-1)h\\ 0 &(a+b-1)^{2}+\rho b^{2} &-(1-\rho)b(a+b-1)\\ 0 &-(1-\rho)b(a+b-1) & b^{2}+\rho(a+b-1)^{2} \end{array}\displaystyle \right ) \left ( \textstyle\begin{array}{@{}c@{}} a_{1}\\ b_{1}\\ c_{1} \end{array}\displaystyle \right ), $$

where $M=(a+b-1)^{2}+b^{2}$.

Using $\rho>0$ and $2(a+b-1)b\leq(a+b-1)^{2}+b^{2}$, we derive the inequality for $|b_{0}|$,

$$\begin{aligned} \vert b_{0} \vert &= \biggl\vert \frac{(a+b-1)^{2}+\rho b^{2}}{(a+b-1)^{2}+b^{2}}b_{1} + \frac {-(1-\rho)b(a+b-1)}{(a+b-1)^{2}+b^{2}}c_{1} \biggr\vert \\ &\leq \biggl\vert \frac{(a+b-1)^{2}+\rho b^{2}}{(a+b-1)^{2}+b^{2}} \biggr\vert \cdot \vert b_{1} \vert + \vert 1-\rho \vert \cdot \biggl\vert \frac{(a+b-1)b}{(a+b-1)^{2}+b^{2}} \biggr\vert \cdot \vert c_{1} \vert \\ &\leq\max\{\rho, 1\}\cdot \vert b_{1} \vert + \frac{1}{2} \vert 1-\rho \vert \cdot \vert c_{1} \vert \\ &\leq\max\biggl\{ \rho, 1, \frac{1}{2} \vert 1-\rho \vert \biggr\} \cdot \bigl( \vert b_{1} \vert + \vert c_{1} \vert \bigr). \end{aligned}$$

Then the inequality for $|c_{0}|$ is derived similarly:

$$ \vert c_{0} \vert \leq\max \biggl\{ \rho, 1, \frac{1}{2} \vert 1-\rho \vert \biggr\} \cdot \bigl( \vert b_{1} \vert + \vert c_{1} \vert \bigr). $$

That is to say, we have

$$ \vert b_{0} \vert + \vert c_{0} \vert \leq2\max \biggl\{ 1, \frac{\beta^{-}}{\beta^{+}}, \frac{1}{2} \biggl\vert 1- \frac{\beta^{-}}{\beta^{+}} \biggr\vert \biggr\} \cdot \bigl( \vert b_{1} \vert + \vert c_{1} \vert \bigr) =C_{1} \bigl( \vert b_{1} \vert + \vert c_{1} \vert \bigr). $$

If we express $a_{1}$, $b_{1}$, $c_{1}$ by $a_{0}$, $b_{0}$, $c_{0}$ by applying (3.11) and (3.12), then we can get the inequalities for $|b_{1}|+|c_{1}|$ similarly,

$$ \vert b_{1} \vert + \vert c_{1} \vert \leq2\max \biggl\{ 1, \frac{\beta^{+}}{\beta^{-}}, \frac{1}{2} \biggl\vert 1- \frac{\beta^{+}}{\beta^{-}} \biggr\vert \biggr\} \cdot \bigl( \vert b_{0} \vert + \vert c_{0} \vert \bigr) =C_{2} \bigl( \vert b_{0} \vert + \vert c_{0} \vert \bigr). $$

Therefore, we also derive the conclusion for Type-2 interface elements. □

For any interface element $T\in\mathcal{T}_{n}^{i}$, we can divide it into four congruent triangles $T_{j}$, $j=1,2,3,4$ by connecting the three midpoints of three edges of *T*. There must exist a subset $T_{j}$ which is completely inside of either $T^{+}$ or $T^{-}$, and we denote it by *T̃*.

If $\widetilde{T}\subset T^{+}$, noting that $|\widetilde{T}|=\frac {|T|}{4}=\frac{1}{8}h^{2}$, we have

$$ \|\nabla\phi\|^{2}_{0,\widetilde{T}}=\bigl(b_{0}^{2}+c_{0}^{2} \bigr)\cdot|\widetilde {T}|=\frac{1}{8}h^{2}\bigl(b_{0}^{2}+c_{0}^{2} \bigr). $$

Applying the left of the estimate (3.23) in the above lemma, we derive

$$ \|\nabla\phi\|^{2}_{0,\widetilde{T}}\geq\frac{1}{16C^{2}_{2}}h^{2} \bigl(b_{1}^{2}+c_{1}^{2}\bigr). $$

So we have the estimate

$$ b_{i}^{2}+c^{2}_{i} \leq16C^{2}_{2}\cdot h^{-2}\|\nabla\phi \|^{2}_{0,\widetilde {T}},\quad i=0, 1. $$

If $\widetilde{T}\subset T^{-}$, by the right of the estimate (3.23), we obtain

$$ b_{i}^{2}+c^{2}_{i} \leq16C^{2}_{1}\cdot h^{-2}\|\nabla\phi \|^{2}_{0,\widetilde {T}}, \quad i=0, 1. $$

Therefore, no matter which part of *T* the subset *T̃* belongs to, we have

3.24 $$ b_{i}^{2}+c^{2}_{i} \leq C h^{-2}\|\nabla\phi\|^{2}_{0,\widetilde{T}},\quad i=0, 1. $$

Now we give the proof for Theorem 3.5.

#### Proof

Without loss of generality, we consider $e=\overline{A_{1}A_{2}}$ with $e^{-}=\overline{A_{1}D}$ and $e^{+}=\overline{DA_{2}}$. Let $\beta_{\max}=\max\{\beta^{-}, \beta^{+}\}$, by the above result (3.24), we obtain

$$\begin{aligned} \Vert \beta\nabla\phi\cdot \mathbf {n}\Vert ^{2}_{0,e}&\leq \beta_{\max}^{2} \bigl( \bigl\Vert \nabla\phi^{-} \bigr\Vert ^{2}_{0,e^{-}}+ \bigl\Vert \nabla\phi^{+} \bigr\Vert ^{2}_{0,e^{+}} \bigr) \\ &\leq\beta_{\max}^{2}h \bigl[\bigl(b_{1}^{2}+c_{1}^{2} \bigr)+\bigl(b_{0}^{2}+c_{0}^{2}\bigr) \bigr] \\ &\leq2C\beta_{\max}^{2}h^{-1} \Vert \nabla\phi \Vert ^{2}_{0,\widetilde {T}} \\ &\leq Ch^{-1} \Vert \nabla\phi \Vert ^{2}_{0,{T}}, \end{aligned}$$

which completes the proof. □

For any $u\in\widetilde{H}_{\mathrm{int}}^{2}(T)$, we let $I_{T}u\in\widehat {S}_{h}(T)$ be such that

$$\int_{e_{j}}I_{T}u \,ds= \int_{e_{j}}u\,ds,\quad j=1,2,3, $$

where $e_{j}$, $j=1,2,3$ are three edges of *T*. We call $I_{T}u$ the interpolant of *u* in $\widehat{S}_{h}(T)$, and we have its error estimate which is stated in [33],

3.25 $$ \|u-I_{T}u\|_{0,T}+h\|u-I_{T}u \|_{1,T}\leq Ch^{2} \|u\|_{2,T}, \quad \forall u\in \widetilde{H}^{2}_{\mathrm{int}}(T), $$

where $\|\cdot\|_{2,T}$ is the norm in $\widetilde{H}^{2}(T)$ defined by (2.5).

We can naturally extend the interpolation operator such that $I_{h}: \widetilde{H}^{2}_{\mathrm{int}}(\Omega)\rightarrow S_{0h}(\Omega)$ and $(I_{h}u)|_{T}=I_{T}(u|_{T})$.

Then, by (3.1) and the above estimation (3.25), we obtain the following result.

#### Theorem 3.7

*For any* $u\in\widetilde{H}^{2}_{\mathrm{int}}(\Omega)$, *there exists a constant* $C>0$ *such that*

$$\|u-I_{h}u\|_{0,\Omega}+h\|u-I_{h}u\|_{1,h} \leq Ch^{2}\|u\|_{\widetilde {H}^{2}(\Omega)}, $$

*where* $\|\cdot\|_{1,h}^{2}=\sum\limits_{T\in\mathcal{T}_{h}}\|\cdot\| _{1,T}^{2}$.

## The Crouzeix-Raviart PIFE method

We multiply the elliptic equation (2.1a) by a test function $v\in S_{0h}(\Omega)$, integrate over each element $T\in\mathcal {T}_{h}$, and apply the Green’s formula,

$$\int_{T}\beta\nabla u \cdot\nabla v \,d\mathbf {x}- \int_{\partial T}(\beta \nabla u \cdot \mathbf {n}_{T})v\,ds= \int_{T} fv\, d\mathbf {x},\quad \forall v\in S_{0h}(\Omega). $$

Here, $\mathbf {n}_{T}$ is the unit outward normal of *T*.

Summing the above equation over all elements, we obtain

4.1 $$ \sum_{T\in\mathcal{T}_{h}} \int_{T}\beta\nabla u \cdot\nabla v \,d\mathbf {x}-\sum _{T\in\mathcal{T}_{h}} \int_{\partial T}(\beta \nabla u \cdot \mathbf {n}_{T})v\,ds= \int_{\Omega}fv\,d\mathbf {x},\quad \forall v\in S_{0h}( \Omega). $$

Let $\mathbf {n}_{e}$ denote the unit normal vector of *e*, then

$$\sum_{T\in\mathcal{T}_{h}} \int_{\partial T}(\beta\nabla u \cdot \mathbf {n}_{T})v\,ds=\sum _{e\in\mathcal{E}^{\circ}_{h}} \int _{e}\bigl[(\beta\nabla u \cdot \mathbf {n}_{e})v\bigr] \,ds+\sum_{e\in\mathcal {E}^{b}_{h}} \int_{e}(\beta\nabla u \cdot \mathbf {n}_{e})v\,ds. $$

Applying the algebraic identity $ac-bd=\frac{1}{2}(a+b)(c-d)+\frac {1}{2}(a-b)(c+d)$ leads to

$$\int_{e}\bigl[(\beta\nabla u \cdot \mathbf {n}_{e})v\bigr] \,ds= \int_{e}\{\beta\nabla u \cdot \mathbf {n}_{e}\}[v]\,ds+ \int_{e}[\beta\nabla u \cdot \mathbf {n}_{e}]\{v\}\,ds. $$

Then substituting the second term in (4.1) by the above two equalities, we obtain

$$\begin{aligned}& \sum_{T\in\mathcal{T}_{h}} \int_{T}\beta\nabla u \cdot\nabla v \,d\mathbf {x}-\sum _{e\in\mathcal{E}^{\circ}_{h}} \int_{e}\{\beta\nabla u \cdot \mathbf {n}_{e}\}[v]\,ds- \sum _{e\in\mathcal{E}^{\circ}_{h}} \int_{e}[\beta\nabla u \cdot \mathbf {n}_{e}]\{v\}\,ds \\& \quad {}-\sum_{e\in\mathcal{E}^{b}_{h}} \int_{e}(\beta\nabla u \cdot \mathbf {n}_{e})v\,ds= \int_{\Omega}fv\,d\mathbf {x}, \quad \forall v\in S_{0h}( \Omega). \end{aligned}$$

Assume that *u* is smooth enough, $\nabla\cdot(\beta\nabla u) = -f \in L^{2}(\Omega)$ implies that $\beta\nabla u \in H(\mathrm{div}; \Omega)$ and thus the normal flux $\beta\nabla u \cdot \mathbf {n}_{e}$ is continuous across every interior edge *e*. Hence, $[\beta\nabla u \cdot \mathbf {n}_{e}]=0$. Therefore the third term in the above equation is zero and the above equation becomes

4.2 $$ \sum_{T\in\mathcal{T}_{h}} \int_{T}\beta\nabla u \cdot\nabla v \,d\mathbf {x}-\sum _{e\in\mathcal{E}_{h}} \int_{e}\{\beta\nabla u \cdot \mathbf {n}_{e}\}[v]\,ds= \int_{\Omega}fv\,d\mathbf {x}, \quad \forall v\in S_{0h}( \Omega), $$

where we have used the definition of the jump and average values for functions on boundary edges and the relationship $\mathcal{E}_{h}= \mathcal{E}^{\circ}_{h}\cup\mathcal{E}^{b}_{h}$.

In the following, similar to [23], we give the partially penalty immersed finite element method with Crouzeix-Raviart elements and prove the existence and uniqueness of its discrete solution. In the derivation of the PIFE formulation, we have followed the idea of the interior penalty discontinuous Galerkin scheme [38]. The meaning of partially penalty is that we add penalty terms only to the interface edges in order to restrict the jump of IFE functions across their interface edges.

Since *u* is continuous in the interior of Ω, $[u]=0$ on each interior edge *e*. We add two penalty terms whose value is zero for the exact solution *u* defined only on interface edges to (4.2), and obtain

4.3 $$\begin{aligned}& \sum_{T\in\mathcal{T}_{h}} \int_{T}\beta\nabla u \cdot\nabla v \,d\mathbf {x}-\sum _{e\in\mathcal{E}_{h}} \int_{e}\{\beta\nabla u \cdot \mathbf {n}_{e}\}[v]\,ds + \varepsilon \sum_{e\in\mathcal {E}^{i}_{h}} \int_{e}\{\beta\nabla v \cdot \mathbf {n}_{e}\}[u]\,ds \\& \quad {}+ \sum_{e\in\mathcal{E}^{i}_{h}} \frac{\sigma^{0}_{e}}{|e|^{\beta _{0}}} \int_{e}[u] [v]\,ds = \int_{\Omega}fv\,d\mathbf {x}, \quad \forall v\in S_{0h}( \Omega). \end{aligned}$$

We assume that on non-interface edges, the quantity $\sum_{e\in\mathcal{E}^{n}_{h}}\int_{e}\{\beta\nabla u \cdot \mathbf {n}_{e}\}[v]\,ds$ is not very large which suggests to ignore this term in our scheme. Then we have

$$\begin{aligned}& \sum_{T\in\mathcal{T}_{h}} \int_{T}\beta\nabla u \cdot\nabla v \,d\mathbf {x}-\sum _{e\in\mathcal{E}^{i}_{h}} \int_{e}\{\beta\nabla u \cdot \mathbf {n}_{e}\}[v]\,ds + \varepsilon \sum_{e\in\mathcal {E}^{i}_{h}} \int_{e}\{\beta\nabla v \cdot \mathbf {n}_{e}\}[u]\,ds \\& \quad {}+ \sum_{e\in\mathcal{E}^{i}_{h}} \frac{\sigma^{0}_{e}}{|e|^{\beta _{0}}} \int_{e}[u] [v]\,ds \approx \int_{\Omega}fv\,d\mathbf {x}, \quad \forall v\in S_{0h}( \Omega), \end{aligned}$$

where we have used the relationship $\mathcal{E}_{h}= \mathcal {E}^{i}_{h}\cup\mathcal{E}^{n}_{h}$.

Now we define the PIFE formulation: find $u_{h}\in S_{0h}(\Omega)$ such that

4.4 $$ a_{\varepsilon }(u_{h},v_{h})=(f,v_{h}), \quad \forall v_{h}\in S_{0h}(\Omega), $$

where

$$\begin{aligned}& a_{\varepsilon }(u_{h},v_{h}) = \sum _{T\in\mathcal{T}_{h}} \int_{T}\beta \nabla u_{h} \cdot\nabla v_{h} \,d\mathbf {x}-\sum_{e\in\mathcal {E}^{i}_{h}} \int_{e}\{\beta\nabla u_{h} \cdot \mathbf {n}_{e} \}[v_{h}]\,ds \\& \hphantom{a_{\varepsilon }(u_{h},v_{h}) ={}}{}+ \varepsilon \sum_{e\in\mathcal{E}^{i}_{h}} \int_{e}\{\beta \nabla v_{h} \cdot \mathbf {n}_{e}\}[u_{h}]\,ds + \sum_{e\in\mathcal{E}^{i}_{h}} \frac{\sigma^{0}_{e}}{|e|^{\beta _{0}}} \int_{e}[u_{h}] [v_{h}]\,ds, \\& (f,v_{h}) = \sum_{T\in\mathcal{T}_{h}} \int_{T}fv_{h}\,d\mathbf {x}. \end{aligned}$$

In the above formulation, the penalty parameter $\sigma_{e}^{0}>0$, the choice of the power $\beta_{0}>0$ will be discussed later, and the parameter *ε* may take the value −1, 0, or 1.

Here, we also call the scheme (4.4) by the same name as reference [23] (shown in Remark 4.1), although the degrees of freedom used are different.

### Remark 4.1

If $\varepsilon =-1$, the scheme (4.4) is called the symmetric PIFE method; if $\varepsilon =+1$, the scheme (4.4) is called the nonsymmetric PIFE method; if $\varepsilon =0$, the scheme (4.4) is called the incomplete PIFE method.

Subsequently, we shall prove the existence and uniqueness of the solution to (4.4). Define the energy norm on $S_{0h}(\Omega)$ by

4.5 $$ \|v\|_{\varepsilon}=\biggl(\sum_{T\in\mathcal{T}_{h}} \int_{T}\beta \nabla v \cdot\nabla v \,d\mathbf {x}+\sum _{e\in\mathcal {E}^{i}_{h}}\frac{\sigma^{0}_{e}}{|e|^{\beta_{0}}} \int_{e}[v] [v] \,ds\biggr)^{1/2},\quad \forall v\in S_{0h}(\Omega). $$

It is easy to check that it is indeed a norm on $S_{0h}(\Omega)$.

In the following theorem, the coercivity of the bilinear form $a_{\varepsilon }(\cdot,\cdot)$ with respect to the energy norm $\|\cdot\| _{\varepsilon}$ is stated, which is similar to that in [23] although the degrees of freedom used are different.

### Theorem 4.2

*The coercivity of the bilinear form* $a_{\varepsilon }(\cdot,\cdot)$:

(I) $a_{+1}$ *is coercive on* $S_{0h}(\Omega)$, *that is*, $a_{+1}(v, v) \geq k\|v\|^{2}_{\varepsilon}$, $\forall v \in S_{0h}(\Omega)$.
(II) $a_{-1}$ *and* $a_{0}$ *are coercive if* $\beta_{0}\geq1$ *and if* $\sigma ^{0}_{e}$ *is bounded below by a constant* $\sigma^{*}_{e}$ *that depends on the diffusion coefficient* *β* *and the constant in the trace inequality*.

### Proof

Note that the coercivity result is trivial for $\varepsilon =1$ and the corresponding coercivity constant is $k=1$. Indeed,

$$a_{+1}(v, v) = \|v\|^{2}_{\varepsilon}, \quad \forall v \in S_{0h}(\Omega). $$

Hence, we focus on the other two cases $\varepsilon =-1$ or 0 below.

For every interface edge $e\in\mathcal{E}_{h}^{i}$ shared by the interface elements $T_{e,1}$ and $T_{e,2}$, using Cauchy-Schwarz’s inequality twice and the trace inequality stated in Theorem 3.5, we derive

$$\begin{aligned} &\int_{e}\{\beta\nabla v \cdot \mathbf {n}_{e}\}[v] \,ds \\ &\quad \leq \bigl\Vert \{\beta\nabla v \cdot \mathbf {n}_{e}\} \bigr\Vert _{0,e}\bigl(|e|^{\beta _{0}}\bigr)^{1/2-1/2} \bigl\Vert [v] \bigr\Vert _{0,e} \\ &\quad \leq\frac{1}{2}{|e|}^{\beta_{0}/2} \bigl( \bigl\Vert (\beta\nabla v \cdot \mathbf {n}_{e})|_{T_{e,1}} \bigr\Vert _{0,e}+ \bigl\Vert (\beta\nabla v \cdot \mathbf {n}_{e})|_{T_{e,2}} \bigr\Vert _{0,e} \bigr)\cdot\frac{1}{|e|^{\beta_{0}/2}} \bigl\Vert [v] \bigr\Vert _{0,e} \\ &\quad \leq\frac{1}{2}|e|^{\beta_{0}/2}\cdot C \bigl(h_{T_{e,1}}^{-1/2} \Vert \sqrt{\beta}\nabla v \Vert _{0,T_{e,1}}+h_{T_{e,2}}^{-1/2} \Vert \sqrt{\beta}\nabla v \Vert _{0,T_{e,2}} \bigr)\cdot \frac {1}{|e|^{\beta_{0}/2}} \bigl\Vert [v] \bigr\Vert _{0,e} \\ &\quad \leq\frac{1}{2}C\cdot\bigl(h_{T_{e,1}}^{\beta _{0}-1}+h_{T_{e,2}}^{\beta_{0}-1} \bigr)^{\frac{1}{2}} \bigl( \Vert \sqrt {\beta}\nabla v \Vert ^{2}_{0,T_{e,1}}+ \Vert \sqrt{\beta}\nabla v \Vert ^{2}_{0,T_{e,2}} \bigr)^{\frac{1}{2}}\cdot\frac{1}{|e|^{\beta _{0}/2}} \bigl\Vert [v] \bigr\Vert _{0,e}, \end{aligned}$$

where $h_{T_{e,1}}$ and $h_{T_{e,2}}$ are the maximum edge length of $T_{e,1}$ and $T_{e,2}$, respectively, and the common edge *e* satisfies $|e|\leq h_{T_{e,1}}$, $|e|\leq h_{T_{e,2}}$.

If $\beta_{0}$ satisfies the condition $\beta_{0}\geq1$ and we assume, without loss of generality, that $h\leq1$. Then, from the above inequality, we obtain

4.6 $$ \int_{e}\{\beta\nabla v \cdot \mathbf {n}_{e}\}[v] \,ds \leq C \bigl( \Vert \sqrt{\beta}\nabla v \Vert _{0,T_{e,1}}^{2}+ \Vert \sqrt{\beta}\nabla v \Vert _{0,T_{e,2}}^{2} \bigr)^{1/2}\cdot\frac {1}{|e|^{\beta_{0}/2}} \bigl\Vert [v] \bigr\Vert _{0,e}. $$

Summing the above (4.6) over all the interface edges, and using Cauchy-Schwarz’s inequality once more and the Young inequality, for $\delta>0$, we get

$$\begin{aligned} &\sum_{e\in\mathcal{E}^{i}_{h}} \int_{e}\{\beta\nabla v \cdot \mathbf {n}_{e}\}[v] \,ds \\ &\quad \leq C \biggl(\sum_{e\in\mathcal{E}^{i}_{h}}\frac{1}{|e|^{\beta _{0}}} \bigl\Vert [v] \bigr\Vert ^{2}_{0,e} \biggr)^{1/2} \biggl(\sum_{e\in \mathcal{E}^{i}_{h}}\bigl( \Vert \sqrt{\beta}\nabla v \Vert _{0,T_{e,1}}^{2}+ \Vert \sqrt{\beta}\nabla v \Vert _{0,T_{e,2}}^{2} \bigr) \biggr)^{1/2} \\ &\quad = C \biggl(\sum_{e\in\mathcal{E}^{i}_{h}}\frac{1}{|e|^{\beta _{0}}} \bigl\Vert [v] \bigr\Vert ^{2}_{0,e} \biggr)^{1/2} \biggl(2\sum_{T\in \mathcal{T}^{i}_{h}} \Vert \sqrt{\beta}\nabla v \Vert _{0,T}^{2} \biggr)^{1/2} \\ &\quad \leq \biggl(\sum_{T\in\mathcal{T}_{h}} \Vert \sqrt{\beta }\nabla v \Vert _{0,T}^{2} \biggr)^{1/2}\cdot\sqrt{2}C \biggl(\sum_{e\in\mathcal{E}^{i}_{h}}\frac{1}{|e|^{\beta_{0}}} \bigl\Vert [v] \bigr\Vert ^{2}_{0,e} \biggr)^{1/2} \\ &\quad \leq\frac{\delta}{2}\sum_{T\in\mathcal{T}_{h}} \Vert \sqrt { \beta}\nabla v \Vert _{0,T}^{2}+\frac{C^{2}}{\delta}\sum _{e\in \mathcal{E}^{i}_{h}}\frac{1}{|e|^{\beta_{0}}} \bigl\Vert [v] \bigr\Vert ^{2}_{0,e}. \end{aligned}$$

Thus, we obtain a lower bound for $a_{\varepsilon }(v,v)$,

$$\begin{aligned} a_{\varepsilon }(v,v) \geq&\sum_{T\in\mathcal{T}_{h}} \int_{T}\beta\nabla v\cdot \nabla v \,d\mathbf {x}+\sum _{e\in\mathcal{E}^{i}_{h}}\frac{\sigma^{0}_{e}}{|e|^{\beta _{0}}} \int_{e}[v] [v] \,ds \\ &-(1-\varepsilon ) \biggl(\frac{\delta}{2}\sum_{T\in\mathcal {T}_{h}} \Vert \sqrt{\beta}\nabla v \Vert _{0,T}^{2}+ \frac{C^{2}}{\delta }\sum_{e\in\mathcal{E}^{i}_{h}}\frac{1}{|e|^{\beta_{0}}} \bigl\Vert [v] \bigr\Vert ^{2}_{0,e} \biggr) \\ =&\biggl(1-\frac{\delta(1-\varepsilon )}{2}\biggr)\sum_{T\in \mathcal{T}_{h}} \Vert \sqrt{\beta}\nabla v \Vert _{0,T}^{2}+ \sum _{e\in\mathcal{E}^{i}_{h}}\frac{\sigma^{0}_{e}(1-\frac {C^{2}(1-\varepsilon )}{\delta\sigma^{0}_{e}})}{|e|^{\beta_{0}}} \bigl\Vert [v] \bigr\Vert ^{2}_{0,e}, \end{aligned}$$

where *C* is the constant in the trace inequality stated in Theorem 3.5.

Choosing *δ* and $\sigma^{0}_{e}$ in the above inequality such that

$$\delta(1-\varepsilon )< 2, \qquad \sigma^{0}_{e}> \frac{C^{2}}{\delta}(1-\varepsilon ), $$

then we have the coercivity result for the cases $\varepsilon =-1$ or 0 as follows:

$$a_{\varepsilon }(v,v)\geq k\|v\|^{2}_{\varepsilon},\quad k=\min \biggl\{ 1-\frac{\delta}{2}(1-\varepsilon ), 1-\frac {C^{2}}{\delta\sigma^{0}_{e}}(1-\varepsilon ) \biggr\} . $$

For instance, $\delta=1$ if $\varepsilon =0$ and $\delta=1/2$ if $\varepsilon =-1$ and choosing $\sigma^{0}_{e}$ large enough (for example, $\sigma^{0}_{e} \geq2C^{2}$ if $\varepsilon =0$ and $\sigma^{0}_{e} \geq8C^{2}$ if $\varepsilon =-1$), then we have the coercivity result with $k=1/2$.

Summarizing the results above, we complete the proof. □

Now we are ready to derive the existence and uniqueness of the PIFE solution as we have done in the paper [23] (which takes values at three vertices as degrees of freedom).

### Theorem 4.3

*Assume that* (I) *or* (II) *holds true*:

(I) *in the nonsymmetric* ($\varepsilon =+1$) *PIFE case*, $\sigma^{0}_{e}>0$ *for all* *e*;
(II) *in the symmetric* ($\varepsilon =-1$) *or incomplete* ($\varepsilon =0$) *PIFE case*, $\beta_{0}\geq1$ *and* $\sigma^{0}_{e}$ *is bounded below by a large constant for all* *e*.

*Then the PIFE solution of the discrete problem* (4.4) *exists and is unique*.

### Proof

Since (4.4) is a linear problem in finite dimension, existence is equivalent to uniqueness. We only have to prove the uniqueness of the PIFE solution.

Assuming that there are two solutions $u^{1}_{h}$ and $u^{2}_{h}$, the difference $w_{h}=u^{1}_{h}-u^{2}_{h}$ satisfies

$$a_{\varepsilon }(w_{h},v_{h})=0, \quad \forall v_{h}\in S_{0h}(\Omega). $$

Taking $v_{h}=w_{h}$, by the coercivity results stated in Theorem 4.2, we have

$$\|w_{h}\|_{\varepsilon}=0. $$

This implies $w_{h}=0$ since $\|\cdot\|_{\varepsilon}$ is a norm on $S_{0h}(\Omega)$, which completes the proof. □

## The convergence analysis of the PIFE method

In this section, we conduct the error estimate for the PIFE solution in the energy norm $\|\cdot\|_{\varepsilon}$. And we firstly give the interpolant error estimate in the energy norm.

### Lemma 5.1

*For* $u\in\widetilde{H}^{2}_{\mathrm{int}}(\Omega)$, *there exists a constant* *C* *independent of* *h* *and interface location*, *such that*

5.1 $$ \|u-I_{h}u\|_{\varepsilon}\leq C \bigl(h+h^{(3-\beta_{0})/2} \bigr)\|u \| _{\widetilde{H}^{2}(\Omega)}. $$

### Proof

$$\begin{aligned} \Vert u-I_{h}u \Vert ^{2}_{\varepsilon}&=\sum _{T\in\mathcal{T}_{h}} \int _{T}\beta\nabla(u-I_{h}u) \cdot \nabla(u-I_{h}u) \,d\mathbf {x}+\sum_{e\in\mathcal{E}^{i}_{h}} \frac{\sigma^{0}_{e}}{|e|^{\beta_{0}}} \int _{e}[u-I_{h}u] [u-I_{h}u] \,ds \\ &\leq\beta_{\max}\sum_{T\in\mathcal {T}_{h}}|u-I_{h}u|^{2}_{1,T} + Ch^{-\beta_{0}}\sum_{e\in\mathcal {E}^{i}_{h}} \bigl\Vert [u-I_{h}u] \bigr\Vert ^{2}_{0,e}, \end{aligned}$$

where we used that $\sigma^{0}_{e}$ is bounded above for any $e\in \mathcal{E}^{i}_{h}$ when it is fixed.

By the interpolant error estimates given in (3.1) and (3.25) as well as the trace inequality (3.20), we obtain

$$\begin{aligned} \|u-I_{h}u\|^{2}_{\varepsilon}&\leq C\sum _{T\in\mathcal{T}_{h}}h^{2}\|u\|^{2}_{2,T} + Ch^{-\beta _{0}}\sum_{T\in\mathcal{T}_{h}}h^{-1} \bigl( \Vert u-I_{h}u \Vert _{0,T}^{2}+h^{2}|u-I_{h}u|^{2}_{1,T} \bigr) \\ &\leq C\sum_{T\in\mathcal{T}_{h}}h^{2}\|u \|^{2}_{2,T} + Ch^{-\beta _{0}}\sum _{T\in\mathcal{T}_{h}}h^{3}\|u\|_{2,T}^{2} \\ &\leq C \bigl(h^{2}+h^{3-\beta_{0}} \bigr)\|u\|^{2}_{\widetilde{H}^{2}(\Omega)}, \end{aligned}$$

which completes the proof. □

### Remark 5.2

The parameter $\beta_{0}$ in the energy norm (4.5) should satisfy $\beta_{0}\leq1$ in order to guarantee that the Crouzeix-Raviart type IFE space ${S}_{0h}(\Omega)$ has the optimal approximation property in the energy norm.

Next, we come to derive the error estimate for the PIFE solution in the energy norm. In the beginning, we give the following lemma which is cited from Lemma 3 in [37], and we shall need it in the proof of the energy norm estimate.

### Lemma 5.3

*Let* *e* *be an edge of* *T*. *Then there exists a constant* $C>0$ *such that for all* $\phi, v\in H^{1}(T)$

$$\biggl\vert \int_{e}\phi(v-\overline{v}_{e})\,ds \biggr\vert \leq Ch|\phi|_{1,T}|v|_{1,T}, $$

*where* $\overline{v}_{e}=\frac{1}{|e|}\int_{e}v\, ds$.

### Theorem 5.4

*Let* $u\in\widetilde{H}^{2}_{\mathrm{int}}(\Omega)$ *and* $u_{h}\in{S}_{0h}(\Omega )$ *be the solutions of the interface problem* (2.1)-(2.2) *and the discrete formulation* (4.4), *respectively*. *Assume* $\beta_{0}=1$, *then there exists a constant* $C>0$ *independent of* *h* *and the location of the interface*, *such that the following optimal energy norm estimate holds*:

5.2 $$ \|u-u_{h}\|_{\varepsilon}\leq Ch\|u \|_{\widetilde{H}^{2}(\Omega)}. $$

### Proof

The equation (4.3) implies that the solution *u* satisfies

$$a_{\varepsilon }(u, v_{h})-\sum_{e\in\mathcal{E}^{n}_{h}} \int_{e}\{ \beta\nabla u \cdot \mathbf {n}_{e} \}[v_{h}]\,ds= \int_{\Omega}fv_{h}\,d\mathbf {x}, \quad \forall v_{h}\in S_{0h}(\Omega). $$

Subtracting (4.4) from the above equation, we obtain

5.3 $$ a_{\varepsilon }(u-u_{h}, v_{h})=\sum _{e\in\mathcal{E}^{n}_{h}} \int_{e}\{ \beta\nabla u \cdot \mathbf {n}_{e} \}[v_{h}]\,ds,\quad \forall v_{h}\in S_{0h}( \Omega). $$

Let $u-u_{h}=(u-I_{h}u)-(u_{h}-I_{h}u)\triangleq\eta-\xi$, then we have

$$a_{\varepsilon }(\xi, v_{h})=a_{\varepsilon }(\eta, v_{h})-\sum_{e\in \mathcal{E}^{n}_{h}} \int_{e}\{\beta\nabla u \cdot \mathbf {n}_{e} \}[v_{h}]\,ds,\quad \forall v_{h}\in S_{0h}( \Omega). $$

Choosing the test function $v_{h}=\xi\in S_{0h}(\Omega)$ and using the coercivity of the bilinear form $a_{\varepsilon }(\cdot,\cdot)$ in Theorem 4.2, we get

5.4 $$\begin{aligned} k\|\xi\|^{2}_{\varepsilon} \leq& a_{\varepsilon }( \xi,\xi)=a_{\varepsilon }(\eta ,\xi)-\sum_{e\in\mathcal{E}^{n}_{h}} \int_{e}\{\beta\nabla u \cdot \mathbf {n}_{e}\}[\xi]\,ds \\ \leq& \biggl\vert \sum_{T\in\mathcal{T}_{h}} \int_{T}\beta\nabla \eta\cdot\nabla\xi \,d\mathbf {x}\biggr\vert + \biggl\vert \sum_{e\in\mathcal {E}^{i}_{h}} \int_{e} \{\beta\nabla\eta\cdot \mathbf {n}_{e}\}[\xi] \,ds \biggr\vert \\ &{}+ \biggl\vert \varepsilon \sum_{e\in\mathcal{E}^{i}_{h}} \int_{e}\{\beta \nabla\xi\cdot \mathbf {n}_{e}\}[\eta] \,ds \biggr\vert \\ &{}+ \biggl\vert \sum_{e\in\mathcal{E}^{i}_{h}}\frac{\sigma ^{0}_{e}}{|e|^{\beta_{0}}} \int_{e}[\eta] [\xi] \,ds \biggr\vert + \biggl\vert \sum _{e\in\mathcal{E}^{n}_{h}} \int_{e}\{\beta\nabla u \cdot \mathbf {n}_{e}\}[\xi ]\,ds \biggr\vert \\ \triangleq& T_{1}+T_{2}+T_{3}+T_{4}+T_{5}. \end{aligned}$$

Using Cauchy-Schwarz’s inequality and Young’s inequality, we have the estimate for $T_{1}$,

$$\begin{aligned} T_{1}&\leq\beta^{1/2}_{\max}\sum _{T\in\mathcal {T}_{h}} \Vert \sqrt{\beta}\nabla\xi \Vert _{0,T} \Vert \nabla\eta \Vert _{0,T} \\ &\leq\beta^{1/2}_{\max} \biggl(\sum _{T\in\mathcal {T}_{h}} \Vert \sqrt{\beta}\nabla\xi \Vert ^{2}_{0,T} \biggr)^{1/2} \biggl(\sum _{T\in\mathcal{T}_{h}} \Vert \nabla\eta \Vert ^{2}_{0,T} \biggr)^{1/2} \\ &\leq\frac{k}{6}\sum_{T\in\mathcal{T}_{h}} \Vert \sqrt { \beta}\nabla\xi \Vert ^{2}_{0,T}+ \frac{3\beta_{\max}}{2k}\sum _{T\in\mathcal{T}_{h}} \Vert \nabla\eta \Vert ^{2}_{0,T} \\ &\leq\frac{k}{6} \Vert \xi \Vert ^{2}_{\varepsilon}+ \frac{3\beta_{\max}}{2k}\sum_{T\in\mathcal {T}_{h}}|\eta|^{2}_{1,T}. \end{aligned}$$

The approximation results (3.1) and (3.25) give the estimate for $T_{1}$,

$$ T_{1}\leq\frac{k}{6}\|\xi\|^{2}_{\varepsilon}+Ch^{2} \|u\|^{2}_{\widetilde {H}^{2}(\Omega)}. $$

Now we bound the term $T_{3}$. By the Cauchy-Schwarz inequality, we have

$$\begin{aligned} T_{3}\leq{}&|\varepsilon | \biggl(\sum_{e\in\mathcal{E}^{i}_{h}} \bigl\Vert \{\beta\nabla\xi\cdot \mathbf {n}_{e}\} \bigr\Vert ^{2}_{0,e} \biggr)^{1/2} \biggl(\sum _{e\in\mathcal{E}^{i}_{h}} \bigl\Vert [\eta] \bigr\Vert ^{2}_{0,e} \biggr)^{1/2} \\ \leq{}& C \biggl(\sum_{e\in\mathcal{E}^{i}_{h}}\bigl( \bigl\Vert (\beta \nabla\xi\cdot \mathbf {n}_{e})|_{T_{e,1}} \bigr\Vert ^{2}_{0,e}+ \bigl\Vert (\beta\nabla \xi\cdot \mathbf {n}_{e})|_{T_{e,2}} \bigr\Vert ^{2}_{0,e} \bigr) \biggr)^{\frac {1}{2}} \\ &{}\times \biggl(\sum_{e\in\mathcal{E}^{i}_{h}}\bigl( \Vert \eta |_{T_{e,1}} \Vert ^{2}_{0,e}+ \Vert \eta|_{T_{e,2}} \Vert ^{2}_{0,e}\bigr) \biggr)^{\frac{1}{2}}, \end{aligned}$$

where $T_{e,1}$ and $T_{e,2}$ share the common edge *e*.

Using the trace inequality stated in Theorem 3.5 for the IFE function *ξ*, we have

$$\begin{aligned} \bigl\Vert (\beta\nabla\xi\cdot \mathbf {n}_{e})|_{T_{e},j} \bigr\Vert ^{2}_{0,e} &\leq Ch^{-1} \Vert \nabla\xi \Vert ^{2}_{0,T_{e,j}} \leq\frac{C}{\beta_{\min}}h^{-1} \Vert \sqrt{\beta}\nabla \xi \Vert ^{2}_{0,T_{e,j}} \\ &\triangleq Ch^{-1} \Vert \sqrt{\beta}\nabla\xi \Vert ^{2}_{0,T_{e,j}}, \quad j=1, 2, \end{aligned}$$

where $\beta_{\min}=\min\{\beta^{-}, \beta^{+}\}$.

And by the trace inequality (3.20) for the $H^{1}$ function *η*, we obtain

5.5 $$ \|\eta|_{T_{e,j}}\|^{2}\leq Ch^{-1} \bigl(\|\eta\|_{0,T_{e,j}}^{2}+h^{2}\| \nabla\eta \|^{2}_{0,T_{e,j}} \bigr), \quad j=1, 2. $$

Then combining with Young’s inequality and approximation result (3.25), we have

$$\begin{aligned} T_{3}&\leq Ch^{-1} \biggl(\sum_{T\in\mathcal{T}^{i}_{h}} \Vert \sqrt{\beta}\nabla\xi \Vert ^{2}_{0,T} \biggr)^{1/2} \biggl(\sum_{T\in\mathcal{T}^{i}_{h}}\bigl( \Vert \eta \Vert _{0,T}^{2}+h^{2}|\eta |^{2}_{1,T}\bigr) \biggr)^{1/2} \\ &\leq\frac{k}{6}\sum_{T\in\mathcal{T}^{i}_{h}} \Vert \sqrt { \beta}\nabla\xi \Vert ^{2}_{0,T}+Ch^{-2} \biggl( \sum_{T\in \mathcal{T}^{i}_{h}}\bigl( \Vert \eta \Vert _{0,T}^{2}+h^{2}|\eta|^{2}_{1,T} \bigr) \biggr) \\ &\leq\frac{k}{6} \Vert \xi \Vert ^{2}_{\varepsilon}+Ch^{2} \Vert u \Vert ^{2}_{\widetilde {H}^{2}(\Omega)}. \end{aligned}$$

The term $T_{4}$ can simply be bounded using Cauchy-Schwarz’s and Young’s inequalities:

$$\begin{aligned} T_{4}&\leq \biggl(\sum_{e\in\mathcal{E}^{i}_{h}} \frac{\sigma ^{0}_{e}}{|e|^{\beta_{0}}} \bigl\Vert [\xi] \bigr\Vert ^{2}_{0,e} \biggr)^{1/2} \biggl(\sum_{e\in\mathcal{E}^{i}_{h}} \frac{\sigma ^{0}_{e}}{|e|^{\beta_{0}}} \bigl\Vert [\eta] \bigr\Vert ^{2}_{0,e} \biggr)^{1/2} \\ &\leq\frac{k}{6}\sum_{e\in\mathcal{E}^{i}_{h}}\frac{\sigma ^{0}_{e}}{|e|^{\beta_{0}}} \bigl\Vert [\xi] \bigr\Vert ^{2}_{0,e}+\frac{3}{2k} \sum_{e\in\mathcal{E}^{i}_{h}}\frac{\sigma^{0}_{e}}{|e|^{\beta_{0}}} \bigl\Vert [\eta] \bigr\Vert ^{2}_{0,e} \\ &\leq\frac{k}{6} \Vert \xi \Vert ^{2}_{\varepsilon}+Ch^{-\beta_{0}} \sum_{e\in\mathcal{E}^{i}_{h}}\bigl( \Vert \eta|_{T_{e,1}} \Vert ^{2}_{0,e}+ \Vert \eta |_{T_{e,2}} \Vert ^{2}_{0,e}\bigr). \end{aligned}$$

Assume $\beta_{0}\leq1$. Using the estimate (5.5) and the approximation result (3.25), we derive

$$\begin{aligned} T_{4}&\leq\frac{k}{6} \Vert \xi \Vert ^{2}_{\varepsilon}+Ch^{-\beta_{0}-1} \sum_{T\in\mathcal{T}^{i}_{h}}\bigl( \Vert \eta \Vert _{0,T}^{2}+h^{2}|\eta |^{2}_{1,T} \bigr) \\ &\leq\frac{k}{6} \Vert \xi \Vert ^{2}_{\varepsilon}+Ch^{3-\beta_{0}} \Vert u \Vert ^{2}_{\widetilde{H}^{2}(\Omega)} \\ &\leq\frac{k}{6} \Vert \xi \Vert ^{2}_{\varepsilon}+Ch^{2} \Vert u \Vert ^{2}_{\widetilde {H}^{2}(\Omega)}. \end{aligned}$$

Next, we come to bound $T_{5}$.

From the definition of the Crouzeix-Raviart type IFE space in (3.18), for $\xi\in S_{0h}(\Omega)$, we have

5.6 $$ \int_{e}[\xi]\,ds=0, \quad \forall e\in\mathcal{E}_{h}, $$

which also implies $\overline{(\xi|_{T_{e,1}})_{e}}=\overline{(\xi |_{T_{e,2}})_{e}}$ for any edge *e* shared by $T_{e,1}$ and $T_{e,2}$.

Note that $\xi|_{T}\in H^{1}(T)$, applying Lemma 5.3 by choosing $\phi=\xi|_{T_{e,j}}-\overline{(\xi|_{T_{e,j}})_{e}}$, $v=\xi |_{T_{e,j}}$, $j=1, 2$, we obtain

$$\begin{aligned} \bigl\Vert [\xi] \bigr\Vert _{0,e}&= \Vert \xi|_{T_{e,1}}- \xi|_{T_{e,2}} \Vert _{0,e} \\ &= \bigl\Vert \bigl(\xi|_{T_{e,1}}-\overline{(\xi|_{T_{e,1}})_{e}} \bigr)+\bigl(\overline{(\xi|_{T_{e,2}})_{e}}-\xi|_{T_{e,2}} \bigr) \bigr\Vert _{0,e} \\ &\leq \bigl\Vert \xi|_{T_{e,1}}-\overline{(\xi|_{T_{e,1}})_{e}} \bigr\Vert _{0,e}+ \bigl\Vert \overline{(\xi|_{T_{e,2}})_{e}}- \xi|_{T_{e,2}} \bigr\Vert _{0,e} \\ &\leq Ch^{1/2}\bigl(|\xi|_{1,T_{e,1}}+|\xi|_{T_{e,2}}\bigr). \end{aligned}$$

Therefore, applying the above estimate and (5.6), combining with the observation that $\beta\nabla u\cdot \mathbf {n}_{e}$ is continuous across any interior edge $e\in\mathcal{E}^{\circ}_{h}$, we obtain

$$\begin{aligned} T_{5}&= \biggl\vert \sum_{e\in\mathcal{E}^{n}_{h}} \int_{e}(\beta\nabla u \cdot \mathbf {n}_{e})[\xi]\,ds \biggr\vert \\ &= \biggl\vert \sum_{e\in\mathcal{E}^{n}_{h}} \int_{e}\bigl(\beta \nabla u \cdot \mathbf {n}_{e}- \overline{(\beta\nabla u \cdot \mathbf {n}_{e})_{e}} \bigr)[\xi]\,ds \biggr\vert \\ &\leq\sum_{e\in\mathcal{E}^{n}_{h}} \bigl\Vert \bigl(\beta\nabla u \cdot \mathbf {n}_{e}-\overline{(\beta\nabla u \cdot \mathbf {n}_{e})_{e}} \bigr) \bigr\Vert _{0,e} \bigl\Vert [\xi] \bigr\Vert _{0,e} \\ &\leq Ch\sum_{T\in\mathcal{T}_{h}} \vert \beta\nabla u \vert _{1,T} \vert \xi \vert _{1,T} \\ &\leq Ch\sum_{T\in\mathcal{T}_{h}} \Vert u \Vert _{2,T} \Vert \nabla\xi \Vert _{0,T}. \end{aligned}$$

Then by using Cauchy-Schwarz’s and Young’s inequalities, we derive

$$\begin{aligned} T_{5}&\leq Ch \biggl(\sum_{T\in\mathcal{T}_{h}} \Vert u \Vert ^{2}_{2,T} \biggr)^{1/2} \biggl(\sum _{T\in\mathcal{T}_{h}} \Vert \sqrt{\beta }\nabla\xi \Vert ^{2}_{0,T} \biggr)^{1/2} \\ &\leq\frac{k}{6} \Vert \xi \Vert ^{2}_{\varepsilon}+Ch^{2} \Vert u \Vert ^{2}_{\widetilde {H}^{2}(\Omega)}. \end{aligned}$$

In order to conclude, it remains to bound the term $T_{2}$. Similarly to what we did to the above terms, applying Cauchy-Schwarz’s inequality and Young’s inequality, we have

$$\begin{aligned} T_{2}&\leq\sum_{e\in\mathcal{E}^{i}_{h}}\biggl( \frac{|e|^{\beta _{0}}}{\sigma^{0}_{e}}\biggr)^{1/2} \bigl\Vert \{\beta\nabla\eta\cdot \mathbf {n}_{e}\} \bigr\Vert _{0,e}\biggl(\frac{\sigma^{0}_{e}}{|e|^{\beta_{0}}} \biggr)^{1/2} \bigl\Vert [\xi] \bigr\Vert _{0,e} \\ &\leq\biggl(\sum_{e\in\mathcal{E}^{i}_{h}}\frac{|e|^{\beta _{0}}}{\sigma^{0}_{e}} \bigl\Vert \{\beta\nabla\eta\cdot \mathbf {n}_{e}\} \bigr\Vert ^{2}_{0,e}\biggr)^{1/2}\biggl(\sum _{e\in\mathcal{E}^{i}_{h}}\frac {\sigma^{0}_{e}}{|e|^{\beta_{0}}} \bigl\Vert [\xi] \bigr\Vert ^{2}_{0,e} \biggr)^{1/2} \\ &\leq\frac{k}{6}\sum_{e\in\mathcal{E}^{i}_{h}}\frac{\sigma ^{0}_{e}}{|e|^{\beta_{0}}} \bigl\Vert [\xi] \bigr\Vert ^{2}_{0,e} +\frac{3}{2k} \sum_{e\in\mathcal{E}^{i}_{h}}\frac{|e|^{\beta _{0}}}{\sigma^{0}_{e}} \bigl\Vert \{\beta \nabla\eta\cdot \mathbf {n}_{e}\} \bigr\Vert ^{2}_{0,e} \\ &\leq\frac{k}{6} \Vert \xi \Vert ^{2}_{\varepsilon}+ \frac{3}{2k}\sum_{e\in\mathcal{E}^{i}_{h}}\frac{|e|^{\beta _{0}}}{\sigma^{0}_{e}} \bigl\Vert \{\beta\nabla\eta\cdot \mathbf {n}_{e}\} \bigr\Vert ^{2}_{0,e} \\ &\leq\frac{k}{6} \Vert \xi \Vert ^{2}_{\varepsilon}+C \sum_{e\in\mathcal{E}^{i}_{h}}\frac{|e|^{\beta_{0}}}{\sigma ^{0}_{e}}\bigl( \bigl\Vert ( \beta\nabla\eta\cdot \mathbf {n}_{e})|_{T_{e,1}} \bigr\Vert ^{2}_{0,e}+ \bigl\Vert (\beta\nabla\eta\cdot \mathbf {n}_{e})|_{T_{e,2}} \bigr\Vert ^{2}_{0,e} \bigr). \end{aligned}$$

Assume $\beta_{0}\geq1$, then we obtain the estimate for $T_{2}$,

$$\begin{aligned} T_{2}&\leq\frac{k}{6}\|\xi\|^{2}_{\varepsilon}+Ch^{1+\beta_{0}}\|u\|^{2}_{\widetilde{H}^{2}(\Omega)} \\ &\leq\frac{k}{6}\|\xi\|^{2}_{\varepsilon}+Ch^{2} \|u\|^{2}_{\widetilde{H}^{2}(\Omega)}, \end{aligned}$$

where we used $\|(\beta\nabla\eta\cdot \mathbf {n}_{e})|_{T_{e,j}} \| ^{2}_{0,e}\leq Ch\|u\|^{2}_{2,T_{e,j}}$, $j=1,2$, which can be obtained similarly to Lemma 4.4 in [34] by using the approximation result (3.25).

Combining these five bounds above, we derive

$$\frac{k}{6}\|\xi\|^{2}_{\varepsilon}\leq Ch^{2}\|u \|^{2}_{\widetilde {H}^{2}(\Omega)}, $$

which implies the energy norm error estimate for *ξ*, that is, $\| \xi\|_{\varepsilon}\leq Ch\|u\|_{\widetilde{H}^{2}(\Omega)}$.

In addition, Remark 5.2 tells us that, for $\beta _{0}\leq1$,

$$\|\eta\|_{\varepsilon}\leq Ch\|u\|_{\widetilde{H}^{2}(\Omega)}. $$

Finally, we complete the proof by the triangle inequality

$$\|u-u_{h}\|_{\varepsilon}\leq\|\eta\|_{\varepsilon}+\|\xi \|_{\varepsilon}. $$

 □

### Remark 5.5

The error estimate in $H^{1}$-seminorm can be derived as follows:

$$ |u-u_{h}|^{2}_{1,h}=\sum _{T\in\mathcal{T}_{h}}|u-u_{h}|_{1,T}^{2}\leq \frac{1}{\beta_{\min}}\|u-u_{h}\|^{2}_{\varepsilon}\leq Ch^{2}\|u\| ^{2}_{\widetilde{H}^{2}(\Omega)}, $$

that is to say,

5.7 $$ |u-u_{h}|_{1,h}\leq Ch\|u\|_{\widetilde{H}^{2}(\Omega)}. $$

To guarantee the optimal-order energy norm estimate (5.2) of the PIFE solutions, the parameter $\beta_{0}$ in our PIFE schemes (4.4) and the energy norm (4.5) should be chosen as $\beta_{0}=1$. Hence, we assume $\beta_{0}=1$ for the numerical experiments in the next section.

## Computational results

In this section, two kinds of numerical experiments are presented to illustrate the validity of our PIFE schemes with Crouzeix-Raviart elements. One kind is for the isotropic elliptic interface problems with piecewise constant coefficients, which we apply to verify our theoretical findings about the error estimate in the energy norm as well as to show the optimal convergence in $L^{2}$ norm. And the other is for the anisotropic elliptic interface problems in which the diffusion coefficients are piecewise symmetric definite-positive matrices. We conduct this kind of numerical experiments to show that the PIFE method proposed in this paper can also be applied to the problems with discontinuous tensor-coefficients, although we only analyze the problems with discontinuous scalar-coefficients for simplicity.

We consider the elliptic interface problem defined by (2.1a) and (2.2) with $\Omega= [-1,1] \times[-1,1]$, Γ being a circle centered at origin $(0,0)$ with radius $r_{0} = {\pi}/{6.28}$. Then the interface curve Γ separates Ω into two sub-domains $\Omega^{-}$ and $\Omega^{+}$ with

$$\Omega^{-} = \bigl\{ (x,y): x^{2}+y^{2} \leq{r_{0}}^{2} \bigr\} . $$

**Figure 3:**
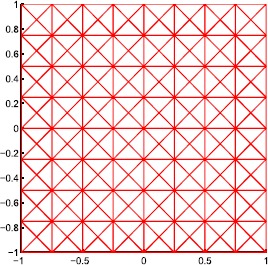
**The triangular partition with** $\pmb{N=8}$ **.**

In our numerical experiments, we use the triangular grids, which are formed by partitioning Ω by $N\times N$ congruent squares and then cutting every square along its two diagonals. Thus we derive our triangulation (illustrated in Figure 3) with mesh size $2/N$, which is the maximum length of edges. The number of triangles in this partition is $N\times N \times4$.

### Numerical results for the isotropic elliptic interface problem

In this subsection, we conduct the numerical experiment with piecewise constant-coefficients to verify our theoretical findings about the PIFE method in symmetric, nonsymmetric and incomplete forms defined in Remark 4.1.

Taking the example from [20], the boundary condition function $g(x,y)$ and the source term $f(x,y)$ are chosen such that the following function *u* is the exact solution:

$$u(x,y)= \textstyle\begin{cases} \frac{1}{\beta^{-}}(x^{2}+y^{2})^{3/2},&(x,y)\in\Omega^{-}, \\ \frac{1}{\beta^{+}}(x^{2}+y^{2})^{3/2}+(\frac{1}{\beta^{-}}-\frac{1}{\beta ^{+}})(\frac{\pi}{6.28})^{3},&(x,y)\in\Omega^{+}. \end{cases} $$

In our computation, we select the penalty parameter $\sigma^{0}_{e}$ in the discrete formulation as $\sigma^{0}_{e}=1$ for the nonsymmetric PIFE scheme, $\sigma^{0}_{e}=10 \max \{\frac{\beta^{-}}{\beta^{+}}, \frac{\beta^{+}}{\beta^{-}} \}$ for the incomplete PIFE scheme and $\sigma^{0}_{e}=100 \max \{\frac{\beta^{-}}{\beta^{+}}, \frac {\beta^{+}}{\beta^{-}} \}$ for the symmetric PIFE scheme.

Then we present the numerical results for $(\beta^{-},\beta ^{+})=(1,1\text{,}000)$ and $(\beta^{-},\beta^{+})=(1\text{,}000,1)$ solved by these three different PIFE schemes, which are reflected by different *ε*, in Table 1 and Table 2, respectively.

**Table 1:** **Numerical results for** $\pmb{(\beta^{-},\beta^{+})=(1,1\text{,}000)}$ **with different PIFE schemes**

**Scheme**	**Grid**	$\boldsymbol{\|u-u_{h}\|_{0,\Omega }}$	**Order**	$\boldsymbol{|u-u_{h}|_{1,h}}$	**Order**
Nonsymmetric PIFE (*ε* = 1)	8 × 8	4.9337E − 3	-	8.0908E − 2	-
	16 × 16	1.1162E − 3	2.1441	4.3830E − 2	0.8844
	32 × 32	2.9009E − 4	1.9440	2.2917E − 2	0.9355
	64 × 64	6.5676E − 5	2.1431	1.1502E − 2	0.9946
	128 × 128	1.8919E − 5	1.7955	5.7744E − 3	0.9941
	256 × 256	4.4585E − 6	2.0852	2.8965E − 3	0.9954
Incomplete PIFE (*ε* = 0)	8 × 8	5.5911E − 3	-	8.1187E − 2	-
	16 × 16	1.3044E − 3	2.0997	4.4172E − 2	0.6087
	32 × 32	3.2578E − 4	2.0014	2.3182E − 2	0.9301
	64 × 64	6.9110E − 5	2.2369	1.1552E − 2	1.0048
	128 × 128	1.8967E − 5	1.8654	5.7761E − 3	1.0000
	256 × 256	4.4796E − 6	2.0820	2.8974E − 3	0.9953
Symmetric PIFE (*ε* = −1)	8 × 8	6.0212E − 3	-	8.1462E − 2	-
	16 × 16	1.3636E − 3	2.1426	4.4097E − 2	0.8854
	32 × 32	3.3744E − 4	2.0147	2.3284E − 2	0.9214
	64 × 64	7.1179E − 5	2.2451	1.1612E − 2	1.0037
	128 × 128	1.8993E − 5	1.9060	5.7769E − 3	1.0073
	256 × 256	4.4914E − 6	2.0802	2.8981E − 3	0.9952

**Table 2:** **Numerical results for** $\pmb{(\beta^{-},\beta^{+})=(1\text{,}000,1)}$ **with different PIFE schemes**

**Scheme**	**Grid**	$\boldsymbol{\|u-u_{h}\|_{0,\Omega }}$	**Order**	$\boldsymbol{|u-u_{h}|_{1,h}}$	**Order**
Nonsymmetric PIFE (*ε* = 1)	8 × 8	1.0678E − 2	-	4.7925E − 1	-
	16 × 16	2.4087E − 3	2.1483	2.3967E − 1	0.9997
	32 × 32	5.7775E − 4	2.0597	1.1989E − 1	0.9993
	64 × 64	1.4172E − 4	2.0274	5.9947E − 2	1.0000
	128 × 128	3.5894E − 5	1.9812	2.9981E − 2	0.9996
	256 × 256	8.8877E − 6	2.0139	1.4993E − 2	0.9998
Incomplete PIFE (*ε* = 0)	8 × 8	1.0510E − 2	-	4.7886E − 1	-
	16 × 16	2.3468E − 3	2.1630	2.3937E − 1	1.0004
	32 × 32	5.7093E − 4	2.0393	1.1983E − 1	0.9982
	64 × 64	1.4131E − 4	2.0144	5.9947E − 2	0.9992
	128 × 128	3.5885E − 5	1.9775	2.9982E − 2	0.9996
	256 × 256	8.8873E − 6	2.0136	1.4993E − 2	0.9998
Symmetric PIFE (*ε* = −1)	8 × 8	1.0501E − 2	-	4.7883E − 1	-
	16 × 16	2.3443E − 3	2.1633	2.3935E − 1	1.0004
	32 × 32	5.7071E − 4	2.0383	1.1984E − 1	0.9981
	64 × 64	1.4128E − 4	2.0142	5.9948E − 2	0.9993
	128 × 128	3.5883E − 5	1.9772	2.9983E − 2	0.9996
	256 × 256	8.8910E − 6	2.0129	1.4993E − 2	0.9998

The data in Table 1 and Table 2 illustrate that, for each scheme, the PIFE solution converges to the exact solution with convergence order $O(h^{2})$ in the $L^{2}$ norm and $O(h)$ in the $H^{1}$-seminorm, which supports our theoretical findings stated in (5.7) and verifies the robustness and validity of this method.

### Numerical results for the anisotropic elliptic interface problem

To show that the PIFE method proposed in this paper can also be applied to anisotropic elliptic interface problems, we provide a numerical example in this subsection, whose diffusion coefficients are piecewise symmetric definite-positive matrices $\mathbb{B}^{l}$, $l=\pm$ satisfying $\mathbb{B}^{+}= t \mathbb {B}^{-}$, $t>0$, $t\neq1$.

The boundary condition function $g(x,y)$ and the source term $f(x,y)$ are chosen such that the exact solution is

$$ u(x,y)= \textstyle\begin{cases} t(x^{2}+y^{2})^{3/2},&(x,y)\in\Omega^{-}, \\ (x^{2}+y^{2})^{3/2}+(t-1)(\frac{\pi}{6.28})^{3},&(x,y)\in\Omega^{+}, \end{cases} $$

which can easily be checked to satisfy the jump conditions (2.2).

In our computation, we select the parameter $\sigma^{0}_{e}$ in the discrete formulation as $\sigma^{0}_{e}=1$ for the nonsymmetric PIFE scheme, $\sigma^{0}_{e}=10 \max\{t, 1/t\}$ for the incomplete PIFE scheme and $\sigma^{0}_{e}=100 \max\{t, 1/t\}$ for the symmetric PIFE scheme.

**Figure 4:**
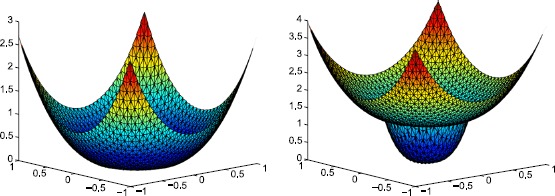
**Numerical solutions under the triangular grid with** $\pmb{N=32}$ **.**

Firstly, we present the numerical results for $t=0.1$, $\mathbb {B}^{-}=[10,20; 20,50]$ and $t=10$, $\mathbb{B}^{-}=[1,2; 2,5]$ solved by all the three PIFE schemes in Table 3 and Table 4, respectively. We can see the optimal-order accuracy in both the $L^{2}$ norm and the $H^{1}$-seminorm, which implies that our PIFE method is valid for the anisotropic interface problem. In addition, we present the figures of the numerical solutions in these two numerical examples (the left one is for $\mathbb{B}^{+}=0.1\mathbb{B}^{-}=[1,2; 2,5]$ and the right one is for $\mathbb{B}^{+}=10\mathbb{B}^{-}=[10,20; 20,50]$), solved by the nonsymmetric ($\varepsilon =1$) PIFE scheme under the triangular grid with $N=32$ (see Figure 4).

**Table 3:** **Numerical results for** $\pmb{\mathbb{B}^{+}=[1,2; 2,5]}$ **,** $\pmb{\mathbb{B}^{-}=[10,20; 20,50]}$

**Scheme**	**Grid**	$\boldsymbol{\|u-u_{h}\|_{0,\Omega }}$	**Order**	$\boldsymbol{|u-u_{h}|_{1,h}}$	**Order**
Nonsymmetric PIFE (*ε* = 1)	8 × 8	1.2388E − 1	-	2.3454	-
	16 × 16	3.1375E − 2	1.9812	1.1815	0.9892
	32 × 32	7.9027E − 3	1.9892	5.9497E − 1	0.9898
	64 × 64	1.9817E − 3	1.9956	2.9851E − 1	0.9950
	128 × 128	4.9509E − 4	2.0010	1.4946E − 1	0.9980
	256 × 256	1.2382E − 4	1.9994	7.4772E − 2	0.9992
Incomplete PIFE (*ε* = 0)	8 × 8	1.2234E − 1	-	2.3267	-
	16 × 16	3.1249E − 2	1.9690	1.1786	0.9812
	32 × 32	7.8952E − 3	1.9848	5.9442E − 1	0.9875
	64 × 64	1.9834E − 3	1.9930	2.9841E − 1	0.9942
	128 × 128	4.9591E − 4	1.9998	1.4941E − 1	0.9980
	256 × 256	1.2407E − 4	1.9989	7.4762E − 2	0.9989
Symmetric PIFE (*ε* = −1)	8 × 8	1.2175E − 1	-	2.3184	-
	16 × 16	3.1205E − 2	1.9640	1.1778	0.9771
	32 × 32	7.8917E − 3	1.9834	5.9433E − 1	0.9867
	64 × 64	1.9836E − 3	1.9923	2.9840E − 1	0.9940
	128 × 128	4.9598E − 4	1.9997	1.4939E − 1	0.9981
	256 × 256	1.2411E − 4	1.9986	7.4760E − 2	0.9988

**Table 4:** **Numerical results for** $\pmb{\mathbb{B}^{+}=[10,20; 20,50]}$ **,** $\pmb{\mathbb{B}^{-}=[1,2; 2,5]}$

**Scheme**	**Grid**	$\boldsymbol{\|u-u_{h}\|_{0,\Omega }}$	**Order**	$\boldsymbol{|u-u_{h}|_{1,h}}$	**Order**
Nonsymmetric PIFE (*ε* = 1)	8 × 8	1.8010E − 1	-	3.5464	-
	16 × 16	5.1864E − 2	1.7960	2.1268	0.7377
	32 × 32	1.4329E − 2	1.8558	1.1824	0.8469
	64 × 64	3.6979E − 3	1.9541	6.1435E − 1	0.9446
	128 × 128	9.5226E − 4	1.9573	3.1524E − 1	0.9626
	256 × 256	2.3939E − 4	1.9920	1.5897E − 1	0.9877
Incomplete PIFE (*ε* = 0)	8 × 8	1.7212E − 1	-	3.3852	-
	16 × 16	5.0836E − 2	1.7595	2.0585	0.7177
	32 × 32	1.4025E − 2	1.8578	1.1467	0.8440
	64 × 64	3.6820E − 3	1.9295	6.0852E − 1	0.9141
	128 × 128	9.5061E − 4	1.9536	3.1442E − 1	0.9526
	256 × 256	2.3906E − 4	1.9915	1.5868E − 1	0.9865
Symmetric PIFE (*ε* = −1)	8 × 8	1.7172E − 1	-	3.3561	-
	16 × 16	5.0887E − 2	1.7547	2.0499	0.7112
	32 × 32	1.4029E − 2	1.8589	1.1439	0.8416
	64 × 64	3.6839E − 3	1.9291	6.0784E − 1	0.9122
	128 × 128	9.5104E − 4	1.9537	3.1429E − 1	0.9516
	256 × 256	2.3912E − 4	1.9918	1.5865E − 1	0.9862

Then we come to verify that our PIFE method can derive the same optimal-order accuracy even if the entries $s^{l}$ of the tensors $\mathbb{B}^{l}$ ($l=\pm$) are negative. We conduct the numerical experiment for $t=0.01$, $\mathbb{B}^{-}=[100,-200; -200,500]$ and $t=100$, $\mathbb{B}^{-}=[1,-2; -2,5]$ and give the error results in Table 5 and Table 6, respectively.

**Table 5:** **Numerical results for** $\pmb{\mathbb{B}^{+}=[1,-2; -2,5]}$ **,** $\pmb{\mathbb{B}^{-}=[100,-200; -200,500]}$

**Scheme**	**Grid**	$\boldsymbol{\|u-u_{h}\|_{0,\Omega }}$	**Order**	$\boldsymbol{|u-u_{h}|_{1,h}}$	**Order**
Nonsymmetric PIFE (*ε* = 1)	8 × 8	1.2304E − 1	-	2.3400	-
	16 × 16	3.1217E − 2	1.9788	1.1799	0.9878
	32 × 32	7.8667E − 3	1.9885	5.9443E − 1	0.9891
	64 × 64	1.9740E − 3	1.9946	2.9836E − 1	0.9944
	128 × 128	4.9317E − 4	2.0010	1.4940E − 1	0.9979
	256 × 256	1.2335E − 4	1.9993	7.4752E − 2	0.9990
Incomplete PIFE (*ε* = 0)	8 × 8	1.2054E − 1	-	2.3118	-
	16 × 16	3.1041E − 2	1.9572	1.1770	0.9739
	32 × 32	7.8555E − 3	1.9824	5.9417E − 1	0.9862
	64 × 64	1.9738E − 3	1.9927	2.9834E − 1	0.9939
	128 × 128	4.9332E − 4	2.0004	1.4937E − 1	0.9981
	256 × 256	1.2343E − 4	1.9988	7.4746E − 2	0.9988
Symmetric PIFE (*ε* = −1)	8 × 8	1.2010E − 1	-	2.3060	-
	16 × 16	3.1019E − 2	1.9530	1.1767	0.9707
	32 × 32	7.8549E − 3	1.9815	5.9414E − 1	0.9858
	64 × 64	1.9737E − 3	1.9927	2.9833E − 1	0.9939
	128 × 128	4.9330E − 4	2.0004	1.4936E − 1	0.9981
	256 × 256	1.2343E − 4	1.9988	7.4746E − 2	0.9987

**Table 6:** **Numerical results for** $\pmb{\mathbb{B}^{+}=[100,-200; -200,500]}$ **,** $\pmb{\mathbb{B}^{-}=[1,-2; -2,5]}$

**Scheme**	**Grid**	$\boldsymbol{\|u-u_{h}\|_{0,\Omega }}$	**Order**	$\boldsymbol {|u-u_{h}|_{1,h}}$	**Order**
Nonsymmetric PIFE (*ε* = 1)	8 × 8	1.0816	-	23.4214	-
	16 × 16	3.9033E − 1	1.4704	16.6163	0.4952
	32 × 32	1.1628E − 1	1.7470	9.8040	0.7612
	64 × 64	3.1365E − 2	1.8904	5.3068	0.8855
	128 × 128	8.2005E − 3	1.9354	2.7690	0.9385
	256 × 256	2.0732E − 3	1.9839	1.4017	0.9822
Incomplete PIFE (*ε* = 0)	8 × 8	1.0751	-	23.1839	-
	16 × 16	3.9068E − 1	1.4603	16.5401	0.4872
	32 × 32	1.1601E − 1	1.7518	9.7531	0.7620
	64 × 64	3.1393E − 2	1.8857	5.2968	0.8808
	128 × 128	8.2042E − 3	1.9360	2.7677	0.9364
	256 × 256	2.0726E − 3	1.9849	1.4011	0.9821
Symmetric PIFE (*ε* = −1)	8 × 8	1.0738	-	23.1231	-
	16 × 16	3.9080E − 1	1.4582	16.5280	0.4844
	32 × 32	1.1596E − 1	1.7528	9.7468	0.7619
	64 × 64	3.1392E − 2	1.8852	5.2953	0.8802
	128 × 128	8.2055E − 3	1.9357	2.7674	0.9362
	256 × 256	2.0730E − 3	1.9848	1.4011	0.9820

Finally, we would like to point out that, even for a larger jump of the diffusion coefficient, our PIFE method is also valid and possesses optimal-order convergence properties. We present numerical results for $t=0.001$, $\mathbb{B}^{-}=[1\text{,}000,2\text{,}000; 2\text{,}000,5\text{,}000]$ in Table 7 as an example for illustration.

**Table 7:** **Numerical results for** $\pmb{\mathbb{B}^{+}=[1,2; 2,5]}$ **,** $\pmb{\mathbb{B}^{-}=[1\text{,}000,2\text{,}000; 2\text{,}000,5\text{,}000]}$

**Scheme**	**Grid**	$\boldsymbol{\|u-u_{h}\|_{0,\Omega }}$	**Order**	$\boldsymbol{|u-u_{h}|_{1,h}}$	**Order**
Nonsymmetric PIFE (*ε* = 1)	8 × 8	1.2074E − 1	-	2.3185	-
	16 × 16	3.1092E − 2	1.9573	1.1779	0.9769
	32 × 32	7.8627E − 3	1.9835	5.9427E − 1	0.9871
	64 × 64	1.9738E − 3	1.9940	2.9834E − 1	0.9942
	128 × 128	4.9306E − 4	2.0011	1.4939E − 1	0.9979
	256 × 256	1.2335E − 4	1.9991	7.4748E − 2	0.9990
Incomplete PIFE (*ε* = 0)	8 × 8	1.1978E − 1	-	2.3039	-
	16 × 16	3.1017E − 2	1.9492	1.1766	0.9695
	32 × 32	7.8636E − 3	1.9798	5.9412E − 1	0.9858
	64 × 64	1.9749E − 3	1.9934	2.9833E − 1	0.9939
	128 × 128	4.9317E − 4	2.0016	1.4936E − 1	0.9981
	256 × 256	1.2339E − 4	1.9989	7.4745E − 2	0.9987
Symmetric PIFE (*ε* = −1)	8 × 8	1.1967E − 1	-	2.3025	-
	16 × 16	3.1010E − 2	1.9482	1.1765	0.9687
	32 × 32	7.8665E − 3	1.9790	5.9412E − 1	0.9857
	64 × 64	1.9752E − 3	1.9937	2.9833E − 1	0.9938
	128 × 128	4.9317E − 4	2.0018	1.4936E − 1	0.9981
	256 × 256	1.2337E − 4	1.9991	7.4745E − 2	0.9987

## Conclusions

In this paper, on non-body fitted triangular meshes, we have developed a partially penalty immersed Crouzeix-Raviart finite element method for both isotropic and anisotropic elliptic interface problems. For the theoretical analysis, we restrict ourselves to the isotropic interface problems for simplicity. The solvability and optimal error estimates are obtained. Then numerical experiments for both isotropic and anisotropic problems are conducted to illustrate the validity of our schemes.

In this method, edge averages on three edges are used as degrees of freedom, which makes it more advantageous for solving anisotropic interface problems than the use of values at three vertices. From numerical examples in the above section, we can observe that it works well for the anisotropic problems, even if the entries $s^{l}$ of the tensor-coefficients $\mathbb{B}^{l}$ ($l=\pm$) are negative, while in [23], by three counter-examples, it is pointed out that, for some specially selected entries of the definite-positive diffusion matrix and the intersection points of the interface with the edges on the interface elements, the piecewise linear Lagrange-nodal-polynomial satisfying the jump conditions cannot be uniquely determined by its values at three vertices.
